# Supplementary material for: Origin, evolution, and distribution of the molecular machinery for biosynthesis of sialylated lipooligosaccharide structures in Campylobacter coli
Source: Sci Rep. 2018 Feb 14;8:3028. doi: 10.1038/s41598-018-21438-2 (PMC5813019; doi:10.1038/s41598-018-21438-2)
Supplement: Supplementary file 1 — Supplemental material 1 [file 41598_2018_21438_MOESM1_ESM.pdf]

Supplementary Table 1

Accession Number NCBI	CstI (Uniprot Q9RGF1)			CstII (Uniprot Q9F0M9)			CstIII (Uniprot Q7BP25)			Blastp BSR Groups
	Max score	Query cover	Ident	Max score	Query cover	Ident	Max score	Query cover	Ident	
WP_075499791.1	594	93%	71%	257	88%	51%	241	82%	50%	Group 1
WP_075479633.1	601	93%	72%	262	88%	52%	239	82%	51%	Group 1
WP_020974326.1	563	93%	62%	261	92%	50%	236	88%	49%	Group 1
WP_078397546.1	591	93%	71%	259	88%	51%	236	82%	50%	Group 1
WP_078397196.1	597	93%	71%	259	88%	51%	236	82%	50%	Group 1
WP_075438134.1	603	93%	73%	258	88%	50%	240	82%	51%	Group 1
WP_075462360.1	594	93%	71%	257	88%	51%	241	82%	50%	Group 1
WP_038854372.1	603	93%	72%	256	92%	50%	244	88%	49%	Group 1
WP_075429285.1	592	93%	70%	254	88%	50%	238	82%	51%	Group 1
WP_075480439.1	584	93%	69%	254	88%	50%	238	82%	51%	Group 1
WP_075447657.1	248	56%	54%	275	98%	50%	471	100%	76%	Group 2
WP_002858290.1	-	-	-	291	98%	53%	592	100%	100%	Group 2
WP_079755069.1	265	61%	51%	272	90%	52%	256	86%	53%	Group 3
WP_002878823.1	274	59%	54%	588	100%	100%	291	95%	53%	Group 4
AHK77095.1	268	59%	53%	537	100%	90%	289	95%	53%	Group 4
WP_057044593.1	271	59%	55%	528	100%	89%	292	95%	53%	Group 4
WP_075456236.1	258	62%	52%	509	98%	87%	292	95%	51%	Group 4
WP_032685951.1	260	62%	52%	508	98%	86%	294	97%	51%	Group 4
WP_072226922.1	260	62%	52%	507	98%	87%	288	95%	51%	Group 4
WP_075485758.1	248	62%	49%	499	99%	85%	289	95%	52%	Group 4
WP_075499417.1	-	-	-	241	98%	49%	202	97%	44%	Group 5
WP_020974593.1	-	-	-	241	98%	48%	201	97%	44%	Group 5
WP_075444400.1	-	-	-	241	98%	48%	201	97%	44%	Group 5
WP_075481154.1	-	-	-	239	98%	48%	200	97%	43%	Group 5
WP_075484527.1	-	-	-	239	98%	48%	199	97%	43%	Group 5
WP_075462815.1	-	-	-	238	98%	48%	200	97%	43%	Group 5
WP_075440629.1	-	-	-	238	98%	48%	200	97%	43%	Group 5
WP_075470126.1	-	-	-	238	98%	48%	201	97%	43%	Group 5
WP_075489678.1	-	-	-	180	99%	37%	155	97%	36%	Group 6
WP_075437196.1	-	-	-	177	99%	38%	149	97%	35%	Group 6
WP_075480001.1	-	-	-	162	95%	36%	139	93%	35%	Group 6
OOX95181.1	-	-	-	202	97%	42%	164	94%	39%	Group 7
WP_075450901.1	-	-	-	197	97%	41%	154	94%	36%	Group 7
WP_078397125.1	-	-	-	196	97%	41%	154	94%	36%	Group 7
WP_075490494.1	-	-	-	194	97%	41%	155	94%	37%	Group 7
WP_075431764.1	-	-	-	194	97%	40%	156	94%	38%	Group 7
WP_002822516.1	-	-	-	190	98%	39%	156	94%	36%	Group 7
WP_002780628.1	-	-	-	190	98%	39%	155	94%	36%	Group 7
WP_053875543.1	-	-	-	189	98%	39%	154	94%	36%	Group 7
WP_075480516.1	*445*	*66%*	*74%*	*266*	*92%*	*50%*	*229*	*88%*	*47%*	*partial sequence*
WP_075503029.1	*476*	*74%*	*71%*	*267*	*92%*	*51%*	*238*	*88%*	*49%*	*partial sequence*
WP_075450515.1	*439*	*66%*	*74%*	*255*	*88%*	*50%*	*236*	*85%*	*49%*	*partial sequence*
WP_075450156.1	*436*	*70%*	*70%*	*254*	*88%*	*50%*	*237*	*82%*	*50%*	*partial sequence*
WP_075460213.1	*427*	*66%*	*72%*	*252*	*92%*	*50%*	*244*	*88%*	*49%*	*partial sequence*
WP_053877509.1	*-*	*-*	*-*	*110*	*79%*	*32%*	*-*	*-*	*-*	*partial sequence*
