# Supplementary material for: Origin, evolution, and distribution of the molecular machinery for biosynthesis of sialylated lipooligosaccharide structures in Campylobacter coli
Source: Sci Rep. 2018 Feb 14;8:3028. doi: 10.1038/s41598-018-21438-2 (PMC5813019; doi:10.1038/s41598-018-21438-2)
Supplement: Supplementary file 2 — Supplemental material 2 [file 41598_2018_21438_MOESM2_ESM.pdf]

upplementary Table 2

Name	Grups	WP_02097	WP_03885	WP_07542	WP_07543	WP_07546	WP_07547	WP_07548	WP_07549	WP_07839	WP_07839	WP_00285	WP_07544	WP_07975	AHK77095	WP_00287	WP_03268	WP_05704	WP_07222	WP_07545	WP_07548	WP_02097	WP_07544	WP_07544	WP_07546	WP_07547	WP_07548	WP_07548	WP_07549	WP_07543	WP_07548	WP_07548	OOX95181	WP_00278	WP_00282	WP_05387	WP_07543	WP_07545	WP_07549	WP_078397125.1
WP_020974326.1	Group 1	100%	73%	74%	74%	73%	74%	74%	73%	74%	73%	25%	27%	28%	27%	28%	27%	28%	27%	27%	27%	18%	18%	18%	18%	18%	18%	18%	19%	15%	13%	14%	15%	15%	15%	15%	15%	15%	16%	15%
WP_038854372.1	Group 1	81%	100%	90%	89%	88%	88%	90%	88%	90%	89%	28%	30%	30%	29%	30%	29%	30%	29%	29%	29%	20%	20%	20%	20%	20%	20%	20%	20%	17%	15%	17%	17%	16%	16%	16%	16%	16%	17%	16%
WP_075429285.1	Group 1	82%	90%	100%	89%	89%	86%	99%	89%	89%	89%	28%	29%	30%	29%	29%	29%	30%	29%	29%	29%	19%	19%	19%	19%	19%	19%	19%	19%	16%	14%	16%	16%	16%	16%	16%	15%	16%	16%	15%
WP_075438134.1	Group 1	80%	89%	89%	100%	95%	89%	89%	95%	88%	90%	28%	30%	29%	30%	30%	29%	30%	29%	29%	30%	20%	20%	20%	20%	20%	20%	20%	20%	16%	14%	16%	16%	16%	16%	16%	15%	16%	16%	15%
WP_075462360.1	Group 1	78%	86%	88%	94%	100%	88%	87%	100%	89%	92%	28%	29%	29%	29%	29%	29%	30%	29%	29%	29%	20%	20%	20%	20%	19%	20%	19%	20%	16%	14%	16%	15%	16%	16%	16%	15%	15%	16%	15%
WP_075479633.1	Group 1	79%	86%	85%	91%	91%	100%	85%	91%	87%	88%	27%	29%	30%	30%	30%	29%	30%	29%	29%	29%	19%	19%	19%	19%	19%	19%	19%	19%	15%	14%	16%	16%	16%	16%	16%	15%	15%	16%	15%
WP_075480439.1	Group 1	82%	90%	99%	89%	89%	86%	100%	89%	89%	88%	28%	29%	30%	29%	30%	29%	30%	29%	29%	29%	19%	19%	19%	19%	19%	19%	19%	19%	16%	14%	16%	16%	16%	16%	16%	15%	16%	16%	15%
WP_075499791.1	Group 1	78%	86%	88%	93%	100%	88%	87%	100%	89%	92%	28%	29%	29%	29%	29%	29%	29%	29%	29%	29%	20%	20%	20%	19%	19%	20%	19%	20%	16%	14%	16%	15%	16%	16%	16%	15%	15%	16%	15%
WP_078397196.1	Group 1	80%	89%	89%	89%	91%	88%	89%	91%	100%	97%	27%	29%	30%	30%	30%	30%	30%	30%	29%	30%	20%	20%	20%	20%	20%	20%	20%	20%	17%	15%	17%	16%	17%	17%	17%	16%	16%	17%	16%
WP_078397546.1	Group 1	80%	89%	89%	91%	93%	89%	89%	93%	97%	100%	28%	29%	30%	30%	30%	30%	30%	30%	30%	30%	20%	20%	20%	20%	20%	20%	20%	20%	17%	15%	17%	17%	17%	17%	17%	16%	16%	17%	16%
WP_002858290.1	Group 2	40%	41%	40%	41%	41%	40%	40%	41%	40%	40%	100%	80%	43%	49%	49%	50%	49%	49%	49%	49%	34%	34%	34%	34%	34%	34%	34%	34%	25%	23%	26%	28%	26%	26%	26%	26%	26%	26%	26%
WP_075447657.1	Group 2	43%	43%	43%	44%	44%	43%	43%	44%	43%	43%	80%	100%	41%	47%	47%	48%	47%	47%	48%	48%	32%	32%	32%	32%	32%	32%	32%	32%	22%	20%	22%	25%	24%	24%	23%	23%	24%	24%	24%
WP_079755069.1	Group 3	41%	41%	41%	41%	41%	41%	41%	41%	41%	41%	41%	38%	100%	44%	43%	43%	44%	43%	43%	42%	31%	30%	31%	30%	30%	31%	30%	31%	22%	20%	22%	25%	26%	26%	25%	24%	25%	25%	24%
AHK77095.1	Group 4	44%	43%	43%	44%	43%	44%	43%	43%	44%	44%	49%	47%	47%	100%	92%	83%	96%	83%	83%	81%	41%	41%	41%	41%	41%	41%	41%	42%	29%	26%	30%	34%	33%	33%	32%	33%	34%	33%	33%
WP_002878823.1	Group 4	44%	44%	43%	44%	44%	45%	43%	44%	44%	44%	49%	47%	46%	91%	100%	86%	90%	86%	87%	85%	41%	40%	41%	40%	40%	41%	41%	41%	30%	28%	31%	34%	32%	32%	32%	33%	34%	33%	33%
WP_032685951.1	Group 4	43%	42%	42%	43%	43%	43%	42%	43%	43%	43%	49%	47%	46%	82%	86%	100%	81%	99%	98%	86%	39%	40%	39%	40%	40%	40%	40%	40%	28%	25%	28%	32%	30%	30%	30%	31%	31%	31%	31%
WP_057044593.1	Group 4	44%	44%	44%	45%	44%	45%	44%	44%	45%	45%	50%	48%	47%	96%	90%	83%	100%	83%	83%	82%	42%	41%	42%	41%	41%	41%	41%	42%	30%	27%	30%	34%	33%	33%	33%	33%	34%	34%	34%
WP_072226922.1	Group 4	43%	43%	42%	43%	43%	44%	42%	43%	43%	43%	49%	47%	46%	82%	86%	99%	82%	100%	99%	86%	40%	40%	40%	40%	40%	40%	40%	40%	28%	24%	27%	33%	30%	30%	30%	31%	32%	32%	31%
WP_075456236.1	Group 4	43%	42%	42%	43%	43%	43%	42%	43%	43%	43%	49%	47%	46%	83%	86%	98%	82%	99%	100%	87%	40%	41%	40%	40%	41%	40%	40%	40%	27%	24%	28%	33%	31%	31%	31%	31%	32%	32%	32%
WP_075485758.1	Group 4	44%	43%	43%	44%	44%	44%	43%	44%	44%	44%	49%	48%	45%	81%	85%	87%	82%	87%	88%	100%	41%	41%	41%	41%	41%	41%	41%	41%	27%	25%	28%	33%	32%	32%	32%	31%	32%	32%	32%
WP_020974593.1	Group 5	29%	29%	28%	28%	28%	28%	28%	29%	29%	29%	33%	31%	32%	40%	40%	39%	40%	39%	39%	40%	100%	98%	100%	98%	97%	99%	98%	100%	37%	35%	37%	41%	39%	39%	39%	40%	40%	40%	40%
WP_075440629.1	Group 5	28%	29%	27%	28%	28%	27%	28%	28%	28%	28%	33%	31%	32%	40%	39%	39%	40%	39%	40%	39%	97%	100%	97%	99%	98%	98%	100%	97%	38%	35%	38%	41%	39%	39%	39%	40%	40%	40%	40%
WP_075444400.1	Group 5	29%	29%	28%	28%	28%	28%	28%	28%	29%	29%	33%	31%	32%	40%	40%	39%	40%	39%	39%	40%	100%	97%	100%	97%	97%	99%	98%	99%	37%	35%	38%	41%	39%	39%	39%	40%	40%	40%	40%
WP_075462815.1	Group 5	28%	29%	28%	28%	28%	28%	28%	28%	29%	29%	33%	31%	32%	40%	39%	39%	40%	39%	40%	39%	98%	100%	98%	100%	99%	98%	99%	97%	38%	35%	38%	41%	39%	40%	39%	40%	40%	41%	40%
WP_075470126.1	Group 5	29%	29%	28%	28%	28%	28%	28%	28%	28%	29%	33%	31%	32%	40%	40%	39%	40%	39%	40%	40%	97%	99%	97%	99%	100%	97%	99%	97%	38%	35%	38%	42%	40%	40%	40%	41%	41%	41%	41%
WP_075481154.1	Group 5	29%	29%	28%	28%	28%	28%	28%	28%	29%	29%	33%	31%	32%	40%	40%	39%	40%	39%	40%	40%	99%	98%	99%	98%	97%	100%	98%	99%	38%	35%	38%	41%	39%	39%	39%	40%	40%	40%	40%
WP_075484527.1	Group 5	28%	28%	27%	28%	28%	27%	27%	28%	28%	28%	33%	31%	31%	40%	39%	39%	40%	39%	39%	39%	98%	100%	97%	99%	98%	98%	100%	97%	38%	35%	38%	41%	39%	39%	39%	40%	40%	40%	40%
WP_075499417.1	Group 5	29%	29%	28%	28%	29%	28%	28%	29%	29%	29%	34%	31%	32%	41%	40%	39%	41%	39%	40%	40%	100%	97%	100%	98%	97%	99%	98%	100%	38%	35%	38%	41%	39%	39%	39%	40%	40%	40%	40%
WP_075437196.1	Group 6	25%	26%	25%	25%	25%	24%	25%	25%	26%	26%	26%	23%	24%	30%	31%	29%	31%	29%	29%	28%	40%	40%	40%	40%	40%	40%	40%	40%	100%	91%	96%	54%	54%	54%	54%	53%	52%	53%	52%
WP_075480001.1	Group 6	22%	24%	22%	22%	22%	22%	22%	22%	23%	23%	26%	21%	23%	29%	30%	27%	29%	26%	27%	27%	39%	39%	39%	39%	39%	39%	39%	38%	95%	100%	100%	55%	54%	54%	55%	53%	53%	54%	53%
WP_075489678.1	Group 6	24%	26%	24%	24%	24%	24%	24%	24%	25%	25%	27%	23%	24%	30%	31%	29%	31%	28%	28%	28%	40%	40%	40%	40%	40%	40%	40%	40%	95%	95%	100%	55%	54%	54%	55%	53%	53%	53%	53%
OOX95181.1	Group 7	23%	24%	22%	23%	22%	23%	22%	22%	23%	23%	27%	24%	25%	33%	33%	31%	33%	31%	32%	32%	41%	40%	41%	40%	41%	40%	40%	41%	50%	49%	51%	100%	76%	77%	76%	94%	94%	93%	95%
WP_002780628.1	Group 7	25%	24%	24%	24%	24%	24%	24%	24%	25%	25%	26%	23%	27%	32%	32%	30%	32%	30%	30%	31%	39%	40%	39%	40%	40%	39%	40%	39%	51%	49%	52%	78%	100%	100%	98%	80%	80%	79%	80%
WP_002822516.1	Group 7	25%	24%	24%	24%	24%	24%	24%	24%	25%	25%	26%	23%	27%	32%	32%	30%	33%	30%	30%	31%	39%	40%	39%	40%	40%	40%	40%	39%	51%	50%	52%	79%	100%	100%	98%	80%	80%	79%	80%
WP_053875543.1	Group 7	25%	24%	24%	24%	24%	24%	24%	24%	25%	25%	26%	23%	27%	32%	32%	30%	32%	30%	30%	31%	39%	39%	39%	40%	40%	39%	40%	39%	52%	50%	52%	78%	98%	98%	100%	78%	79%	78%	79%
WP_075431764.1	Group 7	23%	22%	22%	22%	21%	22%	22%	21%	23%	23%	26%	22%	24%	32%	32%	30%	32%	30%	30%	30%	40%	40%	40%	40%	41%	39%	40%	39%	50%	47%	50%	94%	77%	77%	76%	100%	97%	95%	97%
WP_075450901.1	Group 7	23%	23%	22%	22%	21%	22%	22%	22%	23%	23%	25%	23%	25%	32%	32%	30%	32%	30%	31%	31%	40%	40%	40%	40%	41%	39%	40%	40%	49%	47%	49%	94%	77%	77%	77%	97%	100%	96%	99%
WP_075490494.1	Group 7	24%	24%	23%	23%	23%	23%	23%	23%	24%	24%	25%	23%	25%	32%	32%	30%	32%	30%	31%	31%	40%	40%	40%	40%	41%	40%	40%	40%	49%	48%	50%	93%	76%	76%	75%	95%	96%	100%	96%
WP_078397125.1	Group 7	23%	23%	22%	22%	21%	22%	22%	21%	23%	23%	25%	23%	25%	32%	32%	30%	32%	30%	31%	30%	40%	40%	40%	40%	41%	39%	40%	39%	49%	47%	49%	94%	77%	77%	76%	97%	99%	96%	100%

Supplementary Table 3

Group1	WP_020974326.1	WP_0754796	WP_0783971	WP_0783975	WP_0754381	WP_0754623	WP_0754997	WP_0388543	WP_0754804	WP_075429285.1
WP_020974326.1	100%	74%	74%	73%	74%	73%	73%	73%	74%	74%
WP_075479633.1	79%	100%	87%	88%	91%	91%	91%	86%	85%	85%
WP_078397196.1	80%	88%	100%	97%	89%	91%	91%	89%	89%	89%
WP_078397546.1	80%	89%	97%	100%	91%	93%	93%	89%	89%	89%
WP_075438134.1	80%	89%	88%	90%	100%	95%	95%	89%	89%	89%
WP_075462360.1	78%	88%	89%	92%	94%	100%	100%	86%	87%	88%
WP_075499791.1	78%	88%	89%	92%	93%	100%	100%	86%	87%	88%
WP_038854372.1	81%	88%	90%	89%	89%	88%	88%	100%	90%	90%
WP_075480439.1	82%	86%	89%	88%	89%	89%	89%	90%	100%	99%
WP_075429285.1	82%	86%	89%	89%	89%	89%	89%	90%	99%	100%

Group2	WP_075447657.1	WP_002858290.1
WP_075447657.1	100%	80%
WP_002858290.1	80%	100%

Group3	WP_079755069.1	WP_0570445	AHK77095.1	WP_07222692	WP_03268595	WP_07545623	WP_00287882	WP_07548575	WP_03885437	WP_02097432	WP_07547963	WP_07550302	WP_075480439.1	WP_07542928	WP_07545051
WP_079755069.1	100%	44%	44%	43%	43%	43%	43%	42%	41%	41%	41%	41%	41%	41%	41%

Group3	WP_07546236	WP_07549979	WP_00285829	WP_07546021	WP_07543813	WP_07839719	WP_07839754	WP_07548051	WP_07545015	WP_07544765	WP_07549941	WP_02097459	WP_07544440	WP_07548115	WP_07546281
WP_079755069.1	41%	41%	41%	41%	41%	41%	41%	41%	39%	38%	31%	31%	31%	31%	30%

Group3	WP_07547012	WP_07548452	WP_07544062	WP_00282251	WP_00278062	WP_05387554	WP_07549049	WP_07545090	OOX95181.1	WP_07839712	WP_07543176	WP_07548967	WP_07543719	WP_07548000	WP_053877509.1
WP_079755069.1	30%	30%	30%	26%	26%	25%	25%	25%	25%	24%	24%	22%	22%	20%	1%

Group4	WP_057044593.1	AHK77095.1	WP_0028788	WP_0754857	WP_0754562	WP_0722269	WP_032685951.1
WP_057044593.1	100%	96%	90%	82%	83%	83%	83%
AHK77095.1	96%	100%	92%	81%	83%	83%	83%
WP_002878823.1	90%	91%	100%	85%	87%	86%	86%
WP_075485758.1	82%	81%	85%	100%	88%	87%	87%
WP_075456236.1	82%	83%	86%	87%	100%	99%	98%
WP_072226922.1	82%	82%	86%	86%	99%	100%	99%
WP_032685951.1	81%	82%	86%	86%	98%	99%	100%

Group5	WP_075440629.1	WP_07548452	WP_07546281	WP_07547012	WP_07549941	WP_07544440	WP_07548115	WP_020974593.1
WP_075440629.1	100%	100%	99%	98%	97%	97%	98%	97%
WP_075484527.1	100%	100%	99%	98%	97%	97%	98%	98%
WP_075462815.1	100%	99%	100%	99%	97%	98%	98%	98%
WP_075470126.1	99%	99%	99%	100%	97%	97%	97%	97%
WP_075499417.1	97%	98%	98%	97%	100%	100%	99%	100%
WP_075444400.1	97%	98%	97%	97%	99%	100%	99%	100%
WP_075481154.1	98%	98%	98%	97%	99%	99%	100%	99%
WP_020974593.1	98%	98%	98%	97%	100%	100%	99%	100%

Group6	WP_075437196.1	WP_07548000	WP_075489678.1
WP_075437196.1	100%	91%	96%
WP_075480001.1	95%	100%	100%
WP_075489678.1	95%	95%	100%

Group7	WP_053875543.1	WP_00278062	WP_00282251	OOX95181.1	WP_07549049	WP_07543176	WP_07839712	WP_075450901.1
WP_053875543.1	100%	98%	98%	78%	78%	78%	79%	79%
WP_002780628.1	98%	100%	100%	78%	79%	80%	80%	80%
WP_002822516.1	98%	100%	100%	79%	79%	80%	80%	80%
OOX95181.1	76%	76%	77%	100%	93%	94%	95%	94%
WP_075490494.1	75%	76%	76%	93%	100%	95%	96%	96%
WP_075431764.1	76%	77%	77%	94%	95%	100%	97%	97%
WP_078397125.1	76%	77%	77%	94%	96%	97%	100%	99%
WP_075450901.1	77%	77%	77%	94%	96%	97%	99%	100%

plementary Table 4

Sequence	LOS classes											Cluster A GT-42				Cluster B GT-42			NeuB	Clade
	I	II	III	IV	VI	VII	VIII	IX	X	XI	XII	cstI (G1)	cstIII (G2)	cstVII (G3)	cstII (G4)	cstV (G5)	cstVI (G6)	cstIV (G7)		
ERR083880	0	0	100	0	0	6	0	0	23	17	0	0	0	0	0	0	1	0	0	1
ERR083884	0	0	100	0	0	6	6	0	23	17	0	0	0	0	0	0	1	0	0	1
ERR083912	14	0	9	0	0	0	94	38	0	8	0	0	0	0	1	0	0	0	1	1
ERR083913	0	0	100	0	0	6	6	0	23	17	0	0	0	0	0	0	1	0	0	1
ERR083924	0	0	100	0	0	6	6	0	23	17	0	0	0	0	0	0	1	0	0	1
ERR083928	0	0	100	0	7	6	6	0	23	17	0	0	0	0	0	0	1	0	0	1
ERR083953	0	0	100	0	0	6	6	0	23	17	0	0	0	0	0	0	1	0	0	1
ERR083996	0	0	100	0	0	6	0	0	15	0	0	0	0	0	0	0	1	0	0	1
ERR084002	0	92	0	0	0	0	0	0	0	0	0	0	0	0	0	0	0	1	0	1
ERR084011	14	0	0	0	0	0	6	0	0	0	0	0	0	0	1	0	0	0	1	1
ERR084042	0	0	91	0	0	6	6	0	23	8	0	0	0	0	0	0	1	0	0	1
ERR084055	0	88	0	0	0	0	6	0	0	0	0	0	0	0	0	0	0	1	0	1
ERR084056	0	92	0	0	0	0	6	0	0	0	0	0	0	0	0	0	0	1	0	1
ERR084059	0	0	91	0	0	6	6	0	23	17	0	0	0	0	0	0	1	0	0	1
ERR084071	0	100	0	0	0	0	6	0	0	0	0	0	0	0	0	0	0	1	0	1
ERR084082	0	0	100	0	0	6	6	0	23	8	0	0	0	0	0	0	1	0	0	1
ERR084083	0	0	91	0	0	6	6	0	23	8	0	0	0	0	0	0	1	0	0	1
ERR084085	0	0	100	0	0	6	6	0	23	17	0	0	0	0	0	0	1	0	0	1
ERR084115	0	0	91	0	0	6	6	0	23	8	0	0	0	0	0	0	1	0	0	1
ERR084124	0	0	100	0	0	6	6	0	23	17	0	0	0	0	0	0	1	0	0	1
ERR084134	0	100	0	0	0	0	6	0	0	0	0	0	0	0	0	0	0	1	0	1
ERR084148	0	100	0	0	0	0	6	0	0	0	0	0	0	0	0	0	0	1	0	1
ERR108323	0	100	0	0	0	0	6	0	0	0	0	0	0	0	0	0	0	1	0	1
ERR1147218	0	92	0	0	0	0	6	0	0	0	0	0	0	0	0	0	0	1	0	1
ERR1147219	0	88	0	0	0	0	6	0	0	0	0	0	0	0	0	0	0	1	0	1
ERR1163389	0	0	91	0	0	6	0	0	23	8	0	0	0	0	0	0	1	0	0	1
ERR1163390	0	0	91	0	0	6	0	0	15	8	0	0	0	0	0	0	1	0	0	1
ERR1163391	0	0	91	0	0	6	0	0	23	8	0	0	0	0	0	0	1	0	0	1
ERR1163392	0	96	0	0	0	0	6	0	0	0	0	0	0	0	0	0	0	1	0	1
ERR1163393	0	8	9	0	0	6	0	38	0	0	0	0	0	0	1	0	0	0	1	1
ERR1163397	0	0	91	0	0	6	0	0	15	8	0	0	0	0	0	0	1	0	0	1
ERR1163398	0	0	91	0	0	6	0	0	23	8	0	0	0	0	0	0	1	0	0	1
ERR1163399	0	0	91	0	0	6	0	0	23	8	0	0	0	0	0	0	1	0	0	1
ERR1163400	0	0	100	0	0	6	0	0	23	8	0	0	0	0	0	0	1	0	0	1
ERR1163401	0	0	100	0	0	6	0	0	23	8	0	0	0	0	0	0	1	0	0	1
ERR1163403	0	0	91	0	0	6	6	0	15	8	0	0	0	0	0	0	1	0	0	1
ERR1163404	0	0	100	0	0	6	0	0	15	17	0	0	0	0	0	0	1	0	0	1
ERR1163405	0	0	91	0	0	6	6	0	23	8	0	0	0	0	0	0	1	0	0	1
ERR1163406	0	0	100	0	0	6	0	0	23	8	0	0	0	0	0	0	1	0	0	1
ERR1163407	0	0	91	0	0	6	0	0	23	8	0	0	0	0	0	0	1	0	0	1
ERR1163410	0	0	91	0	0	6	6	0	23	8	0	0	0	0	0	0	1	0	0	1
ERR1163412	0	0	100	0	0	6	0	0	23	8	0	0	0	0	0	0	1	0	0	1
ERR1163415	0	0	100	0	0	6	0	0	23	8	0	0	0	0	0	0	1	0	0	1
ERR1163418	0	0	100	0	0	6	6	0	23	8	0	0	0	0	0	0	1	0	0	1
ERR1163419	0	0	91	0	0	6	6	0	23	8	0	0	0	0	0	0	1	0	0	1
ERR1163420	0	0	100	0	0	6	0	0	23	8	0	0	0	0	0	0	1	0	0	1
ERR1163421	0	0	100	0	0	6	0	0	23	8	0	0	0	0	0	0	1	0	0	1
ERR1163422	0	0	100	0	0	6	0	0	23	8	0	0	0	0	0	0	1	0	0	1
ERR1163423	0	0	100	0	0	6	0	0	23	8	0	0	0	0	0	0	1	0	0	1
ERR1163426	0	0	91	0	0	6	6	0	15	8	0	0	0	0	0	0	1	0	0	1
ERR1163428	0	0	100	0	0	6	6	0	23	8	0	0	0	0	0	0	1	0	0	1
ERR1163429	0	0	100	0	0	6	0	0	23	8	0	0	0	0	0	0	1	0	0	1
ERR1163430	0	0	100	0	0	6	0	0	23	8	0	0	0	0	0	0	1	0	0	1
ERR1163431	0	0	100	0	0	6	0	0	23	8	0	0	0	0	0	0	1	0	0	1
ERR1163435	0	0	91	0	0	6	19	0	23	8	0	0	0	0	0	0	1	0	0	1
ERR1163436	0	0	100	0	0	6	0	0	23	8	0	0	0	0	0	0	1	0	0	1
ERR1163437	0	0	100	0	0	6	0	0	23	8	0	0	0	0	0	0	1	0	0	1
ERR1163438	0	0	100	0	0	6	0	0	23	8	0	0	0	0	0	0	1	0	0	1
ERR1163439	0	0	100	0	0	6	0	0	23	8	0	0	0	0	0	0	1	0	0	1
ERR1163440	0	0	100	0	0	6	6	0	23	8	0	0	0	0	0	0	1	0	0	1
ERR1163442	0	0	100	0	7	6	0	0	23	8	0	0	0	0	0	0	1	0	0	1
ERR1163444	0	0	100	0	0	6	0	0	23	8	0	0	0	0	0	0	1	0	0	1
ERR1163445	0	0	100	0	0	6	0	0	23	8	0	0	0	0	0	0	1	0	0	1
ERR1163446	0	0	91	0	0	6	0	0	23	8	0	0	0	0	0	0	1	0	0	1
ERR1163447	0	0	100	0	0	6	0	0	23	8	0	0	0	0	0	0	1	0	0	1
ERR1163451	0	8	9	0	0	6	0	38	0	0	0	0	0	0	1	0	0	0	1	1
ERR1163452	0	0	100	0	0	6	0	0	23	8	0	0	0	0	0	0	1	0	0	1
ERR1163453	0	0	91	0	0	6	0	0	23	8	0	0	0	0	0	0	1	0	0	1
ERR1163454	0	0	100	0	0	6	0	0	23	8	0	0	0	0	0	0	1	0	0	1
ERR1163455	0	0	100	0	0	6	0	0	23	8	0	0	0	0	0	0	1	0	0	1
ERR1163458	0	0	91	0	0	6	0	0	23	8	0	0	0	0	0	0	1	0	0	1
ERR1163460	0	0	91	0	0	6	0	0	23	8	0	0	0	0	0	0	1	0	0	1

ERR1163461	0	0	91	0	0	6	0	0	23	8	0	0	0	0	0	0	1	0	0	1
ERR1163462	0	0	91	0	0	6	0	0	23	8	0	0	0	0	0	0	1	0	0	1
ERR1163463	0	0	100	0	0	6	0	0	23	8	0	0	0	0	0	0	1	0	0	1
ERR1163468	0	0	100	0	0	6	0	0	23	8	0	0	0	0	0	0	1	0	0	1
ERR1163469	0	0	91	0	0	6	0	0	23	8	0	0	0	0	0	0	1	0	0	1
ERR1163470	0	0	100	0	0	6	0	0	23	8	0	0	0	0	0	0	1	0	0	1
ERR1163471	0	0	100	0	0	6	0	0	23	8	0	0	0	0	0	0	1	0	0	1
ERR1163472	0	0	100	0	0	6	6	0	23	8	0	0	0	0	0	0	1	0	0	1
ERR1163474	0	0	100	0	0	6	0	0	23	17	0	0	0	0	0	0	1	0	0	1
ERR1163475	0	0	100	0	0	6	0	0	23	8	0	0	0	0	0	0	1	0	0	1
ERR1163476	0	0	91	0	0	6	0	0	15	8	0	0	0	0	0	0	1	0	0	1
ERR1163477	0	96	0	0	0	0	6	0	0	0	0	0	0	0	0	0	0	1	0	1
ERR1163478	0	0	100	0	0	6	0	0	15	8	0	0	0	0	0	0	1	0	0	1
ERR1163479	0	0	91	0	0	6	0	0	23	8	0	0	0	0	0	0	1	0	0	1
ERR1163480	0	0	91	0	0	6	0	0	23	8	0	0	0	0	0	0	1	0	0	1
ERR1163481	0	0	91	0	0	6	0	0	23	8	0	0	0	0	0	0	1	0	0	1
ERR1163482	0	0	91	0	0	6	0	0	23	8	0	0	0	0	0	0	1	0	0	1
ERR1163483	0	0	100	0	0	6	0	0	23	8	0	0	0	0	0	0	1	0	0	1
ERR1163484	0	0	91	0	0	6	0	0	23	8	0	0	0	0	0	0	1	0	0	1
ERR1163485	0	0	100	0	0	6	0	0	23	8	0	0	0	0	0	0	1	0	0	1
ERR1163486	0	0	100	0	0	6	0	0	23	8	0	0	0	0	0	0	1	0	0	1
ERR1163487	0	0	91	0	0	6	0	0	15	8	0	0	0	0	0	0	1	0	0	1
ERR1163488	0	0	91	0	0	6	0	0	23	8	0	0	0	0	0	0	1	0	0	1
ERR1163490	0	0	91	0	0	6	0	0	8	8	0	0	0	0	0	0	1	0	0	1
ERR1163491	0	0	91	0	0	6	0	0	8	8	0	0	0	0	0	0	1	0	0	1
ERR1163492	0	0	91	0	0	6	0	0	23	8	0	0	0	0	0	0	1	0	0	1
ERR1163495	0	0	100	0	0	6	0	0	15	8	0	0	0	0	0	0	1	0	0	1
ERR1163496	0	0	100	0	0	6	0	0	23	8	0	0	0	0	0	0	1	0	0	1
ERR1163497	0	0	91	0	0	6	0	0	23	8	0	0	0	0	0	0	1	0	0	1
ERR1163498	0	0	91	0	0	6	0	0	23	8	0	0	0	0	0	0	1	0	0	1
ERR1163499	0	0	91	0	0	6	0	0	15	8	0	0	0	0	0	0	1	0	0	1
ERR1163500	0	0	100	0	0	6	0	0	23	8	0	0	0	0	0	0	1	0	0	1
ERR1163501	0	0	91	0	0	6	0	0	23	8	0	0	0	0	0	0	1	0	0	1
ERR1163502	0	0	100	0	0	6	0	0	23	8	0	0	0	0	0	0	1	0	0	1
ERR1163503	0	0	91	0	0	6	0	0	23	8	0	0	0	0	0	0	1	0	0	1
ERR1163504	0	0	100	0	0	6	0	0	23	8	0	0	0	0	0	0	1	0	0	1
ERR1163505	0	0	91	0	0	6	0	0	23	8	0	0	0	0	0	0	1	0	0	1
ERR1163506	0	0	91	0	0	6	0	0	23	8	0	0	0	0	0	0	1	0	0	1
ERR1163507	0	0	91	0	0	6	0	0	8	8	0	0	0	0	0	0	1	0	0	1
ERR1163509	0	0	91	0	0	6	0	0	15	8	0	0	0	0	0	0	1	0	0	1
ERR1163510	0	0	91	0	0	6	0	0	23	8	0	0	0	0	0	0	1	0	0	1
ERR1163511	0	0	91	0	0	6	0	0	23	8	0	0	0	0	0	0	1	0	0	1
ERR1163512	0	0	100	0	0	6	0	0	23	8	0	0	0	0	0	0	1	0	0	1
ERR1163513	0	0	100	0	0	6	6	0	23	8	0	0	0	0	0	0	1	0	0	1
ERR1163515	0	0	100	0	0	6	0	0	23	8	0	0	0	0	0	0	1	0	0	1
ERR1163516	0	0	100	0	0	6	0	0	23	8	0	0	0	0	0	0	1	0	0	1
ERR1163517	0	0	91	0	0	6	0	0	23	8	0	0	0	0	0	0	1	0	0	1
ERR1163518	0	0	100	0	0	6	0	0	23	8	0	0	0	0	0	0	1	0	0	1
ERR1163519	0	0	100	0	0	6	0	0	23	8	0	0	0	0	0	0	1	0	0	1
ERR1163520	0	0	100	0	0	6	0	0	23	8	0	0	0	0	0	0	1	0	0	1
ERR1163521	0	0	91	0	0	6	0	0	23	8	0	0	0	0	0	0	1	0	0	1
ERR1163522	0	0	100	0	0	6	0	0	23	8	0	0	0	0	0	0	1	0	0	1
ERR1163523	0	0	91	0	0	6	0	0	15	8	0	0	0	0	0	0	1	0	0	1
ERR1163524	0	0	91	0	0	6	0	0	23	8	0	0	0	0	0	0	1	0	0	1
ERR1163525	0	0	100	0	0	6	0	0	23	8	0	0	0	0	0	0	1	0	0	1
ERR1163526	0	0	91	0	0	6	0	0	23	8	0	0	0	0	0	0	1	0	0	1
ERR1163527	0	0	100	0	0	6	0	0	23	8	0	0	0	0	0	0	1	0	0	1
ERR1163528	0	0	91	0	0	6	0	0	23	8	0	0	0	0	0	0	1	0	0	1
ERR1163529	0	0	91	0	0	6	0	0	23	8	0	0	0	0	0	0	1	0	0	1
ERR1163530	0	0	100	0	0	6	0	0	23	8	0	0	0	0	0	0	1	0	0	1
ERR1163531	0	0	100	0	0	6	0	0	23	8	0	0	0	0	0	0	1	0	0	1
ERR1163532	0	0	100	0	0	6	0	0	23	8	0	0	0	0	0	0	1	0	0	1
ERR1163533	0	0	91	0	0	6	0	0	23	17	0	0	0	0	0	0	1	0	0	1
ERR1163534	0	0	100	0	0	6	0	0	23	8	0	0	0	0	0	0	1	0	0	1
ERR1163535	0	0	91	0	0	6	0	0	23	8	0	0	0	0	0	0	1	0	0	1
ERR1163536	0	0	91	0	0	6	0	0	23	8	0	0	1	0	0	0	1	0	1	1
ERR1163537	0	0	91	0	0	6	0	0	23	8	0	0	0	0	0	0	1	0	0	1
ERR1163538	0	0	91	0	0	6	0	0	23	8	0	0	0	0	0	0	1	0	0	1
ERR1163539	0	0	91	0	0	6	0	0	23	8	0	0	0	0	0	0	1	0	0	1
ERR1163540	0	0	100	0	0	6	0	0	23	8	0	0	0	0	0	0	1	0	0	1
ERR1163541	0	0	91	0	0	6	0	0	23	8	0	0	0	0	0	0	1	0	0	1
ERR1163542	0	0	100	0	0	6	0	0	23	8	0	0	0	0	0	0	1	0	0	1
ERR1163547	0	0	91	67	47	24	0	38	31	42	0	0	0	0	0	0	1	0	1	1
ERR1163556	0	100	0	0	0	0	0	38	0	0	0	0	0	0	0	0	0	1	1	1
ERR1163573	0	0	0	0	0	0	0	38	0	8	0	0	0	0	1	0	0	0	1	1

ERR1163574	0	0	91	0	0	6	0	0	8	8	0	0	0	0	0	0	1	0	0	1
ERR1163575	0	0	91	0	0	6	0	0	8	8	0	0	0	0	0	0	1	0	0	1
ERR1163576	0	0	91	0	0	0	0	0	8	8	0	0	0	0	0	0	1	0	0	1
ERR1163577	0	0	91	0	0	6	0	0	8	8	0	0	0	0	0	0	1	0	0	1
ERR1163578	0	0	100	0	0	0	0	0	8	8	0	0	0	0	0	0	1	0	0	1
ERR1163579	0	0	91	0	0	6	0	0	8	8	0	0	0	0	0	0	1	0	0	1
ERR1163580	0	0	91	0	0	6	0	0	8	8	0	0	0	0	0	0	1	0	0	1
ERR1163581	0	0	91	0	0	6	0	0	8	8	0	0	0	0	0	0	1	0	0	1
ERR1163582	0	0	91	0	0	0	0	0	0	8	0	0	0	0	0	0	1	0	0	1
ERR1163583	0	0	91	0	0	0	0	0	8	8	0	0	0	0	0	0	1	0	0	1
ERR1163584	0	0	73	0	0	6	0	0	8	8	0	0	0	0	0	0	1	0	0	1
ERR1163585	0	0	91	0	0	0	0	0	8	8	0	0	0	0	0	0	1	0	0	1
ERR1163587	0	0	82	0	0	0	0	0	8	8	0	0	0	0	0	0	1	0	0	1
ERR1163588	0	0	91	0	0	6	0	0	8	8	0	0	0	0	0	0	1	0	0	1
ERR1163589	0	0	91	0	0	6	0	0	8	8	0	0	0	0	0	0	1	0	0	1
ERR1163590	0	0	91	0	0	6	0	0	8	8	0	0	0	0	0	0	1	0	0	1
ERR1163591	0	0	91	0	0	0	0	0	0	8	0	0	0	0	0	0	1	0	0	1
ERR1163610	14	100	0	0	0	0	6	0	0	0	0	0	0	0	0	0	0	1	0	1
ERR1163618	0	0	91	0	0	6	6	0	15	8	0	0	0	0	0	0	1	0	0	1
ERR1163619	0	0	91	0	0	6	6	0	15	8	0	0	0	0	0	0	1	0	0	1
ERR1163620	0	0	91	0	0	6	6	0	23	8	0	0	0	0	0	0	1	0	0	1
ERR1163621	0	0	91	0	0	6	6	0	8	8	0	0	0	0	0	0	1	0	0	1
ERR1163623	0	0	91	0	0	6	0	38	15	8	0	0	0	0	0	0	1	0	1	1
ERR1163625	0	0	91	0	0	0	0	0	8	8	0	0	0	0	0	0	1	0	0	1
ERR1163627	0	0	82	0	0	6	0	0	8	8	0	0	0	0	0	0	1	0	0	1
ERR1163633	0	0	82	0	0	0	0	0	0	8	0	0	0	0	0	0	1	0	0	1
ERR1163634	0	96	0	0	0	0	6	0	0	0	0	0	0	0	0	0	0	1	0	1
ERR1163635	0	0	91	0	0	6	0	0	15	8	0	0	0	0	0	0	1	0	0	1
ERR1163637	0	0	91	0	0	6	6	0	8	8	0	0	0	0	0	0	1	0	0	1
ERR1163641	0	0	91	0	0	6	0	0	8	8	0	0	0	0	0	0	1	0	0	1
ERR1163642	0	0	91	0	0	0	0	0	0	8	0	0	0	0	0	0	1	0	0	1
ERR1163644	0	0	91	0	0	6	6	0	8	8	0	0	0	0	0	0	1	0	0	1
ERR1163646	29	0	64	0	0	0	19	0	8	8	0	0	0	0	0	0	1	0	0	1
ERR1163648	0	0	91	0	0	6	0	0	8	8	0	0	0	0	0	0	1	0	0	1
ERR1163649	0	0	0	7	0	0	6	38	0	0	0	0	0	0	1	0	0	0	1	1
ERR1163654	0	96	0	0	0	0	0	38	0	0	0	1	0	0	0	0	0	1	1	1
ERR1163656	0	88	0	0	0	0	6	0	0	0	0	0	0	0	0	0	0	1	0	1
ERR1163663	0	0	91	0	0	6	0	0	8	8	0	0	0	0	0	0	1	0	0	1
ERR1163664	0	88	0	0	0	0	6	0	0	0	0	0	0	0	0	0	0	1	0	1
ERR1163665	0	0	91	0	0	6	6	0	23	8	0	0	0	0	0	0	1	0	0	1
ERR1163667	0	0	91	0	0	6	6	0	15	8	0	0	0	0	0	0	1	0	0	1
ERR1163671	0	0	91	0	0	6	0	0	8	8	0	0	0	0	0	0	1	0	0	1
ERR1163673	0	0	91	0	0	6	6	0	15	8	0	0	0	0	0	0	1	0	0	1
ERR1163674	0	0	91	0	0	6	6	0	8	8	0	0	0	0	0	0	1	0	0	1
ERR1163675	0	0	91	0	0	6	6	0	8	8	0	0	0	0	0	0	1	0	0	1
ERR1163678	0	0	91	0	0	6	0	0	8	8	0	0	0	0	0	0	1	0	0	1
ERR1163681	0	0	91	0	0	6	6	0	8	8	0	0	0	0	0	0	1	0	0	1
ERR1163682	0	88	0	0	0	0	6	0	0	0	0	0	0	0	0	0	0	1	0	1
ERR1163683	0	0	91	0	0	6	6	0	23	8	0	0	0	0	0	0	1	0	0	1
ERR1163686	0	0	82	0	0	6	0	0	8	8	0	0	0	0	0	0	1	0	0	1
ERR1163687	0	0	91	0	0	6	0	0	15	8	0	0	0	0	0	0	1	0	0	1
ERR1163688	0	0	91	0	0	6	0	0	8	8	0	0	0	0	0	0	1	0	0	1
ERR1163689	0	0	91	0	0	6	6	0	15	8	0	0	0	0	0	0	1	0	0	1
ERR1163690	0	0	91	0	0	6	6	0	15	8	0	0	0	0	0	0	1	0	0	1
ERR1163691	0	0	91	0	0	6	6	0	23	8	0	0	0	0	0	0	1	0	0	1
ERR1163692	0	0	91	0	0	6	0	0	23	8	0	0	0	0	0	0	1	0	0	1
ERR1163694	0	0	91	0	0	6	0	0	23	8	0	0	0	0	0	0	1	0	0	1
ERR1163695	0	0	91	0	0	6	0	0	8	8	0	0	0	0	0	0	1	0	0	1
ERR1163696	0	0	91	0	0	6	0	0	23	8	0	0	0	0	0	0	1	0	0	1
ERR1163701	0	0	91	0	0	6	0	0	15	8	0	0	0	0	0	0	1	0	0	1
ERR1163702	0	96	0	0	0	0	6	0	0	0	0	0	0	0	0	0	0	1	0	1
ERR1163703	0	0	91	0	0	6	6	0	23	8	0	0	0	0	0	0	1	0	0	1
ERR1163704	0	0	91	0	0	6	6	0	15	8	0	0	0	0	0	0	1	0	0	1
ERR1163705	0	0	91	0	0	6	6	0	15	8	0	0	0	0	0	0	1	0	0	1
ERR1163706	0	0	91	0	0	6	0	0	8	8	0	0	0	0	0	0	1	0	0	1
ERR1163709	0	0	100	0	0	6	0	0	23	8	0	0	0	0	0	0	1	0	0	1
ERR1163714	0	0	91	0	0	6	6	0	23	8	0	0	0	0	0	0	1	0	0	1
ERR1163715	0	0	91	0	0	6	0	0	8	8	0	0	0	0	0	0	1	0	0	1
ERR1163716	0	0	73	0	0	0	0	0	8	8	0	0	0	0	0	0	1	0	0	1
ERR1163719	0	0	91	0	0	6	0	0	15	8	0	0	0	0	0	0	1	0	0	1
ERR1163722	0	100	0	0	0	0	6	0	0	0	0	0	0	0	0	0	0	1	0	1
ERR1163729	0	0	100	0	0	6	0	0	23	8	0	0	0	0	0	0	1	0	0	1
ERR1163730	0	96	0	0	0	0	6	0	0	0	0	0	0	0	0	0	0	1	0	1
ERR1163734	0	0	100	0	0	6	0	0	23	8	0	0	0	0	0	0	1	0	0	1
ERR1163741	0	0	91	0	0	6	6	0	8	8	0	0	0	0	0	0	1	0	0	1

ERR1163748	0	0	91	0	0	6	0	0	23	8	0	0	0	0	0	0	1	0	0	1
ERR1163753	0	0	91	0	0	6	6	0	23	8	0	0	0	0	0	0	1	0	0	1
ERR1163758	0	100	0	0	0	0	0	38	0	0	0	1	0	0	0	0	0	1	1	1
ERR1163769	0	0	100	0	7	6	6	0	23	8	0	0	0	0	0	0	1	0	0	1
ERR1163771	0	0	91	0	0	6	6	0	23	8	0	0	0	0	0	0	1	0	0	1
ERR1163772	0	0	91	0	0	6	6	0	23	8	0	0	0	0	0	0	1	0	0	1
ERR1163774	0	0	91	0	0	6	6	0	23	8	0	0	0	0	0	0	1	0	0	1
ERR1163776	0	0	91	0	0	6	6	0	23	8	0	0	0	0	0	0	1	0	0	1
ERR1163777	0	0	91	0	0	6	0	0	23	8	0	0	0	0	0	0	1	0	0	1
ERR1163783	0	0	100	0	0	6	6	0	23	8	0	0	0	0	0	0	1	0	0	1
ERR1163789	0	0	100	0	0	6	0	0	23	8	0	0	0	0	0	0	1	0	0	1
ERR1163790	0	0	91	0	0	6	0	0	23	8	0	0	0	0	0	0	1	0	0	1
ERR1163800	0	0	91	0	0	12	0	0	23	8	0	0	0	0	0	0	1	0	0	1
ERR1163802	71	8	0	0	0	0	0	13	0	0	0	0	0	1	0	0	0	0	1	1
ERR1163805	0	84	0	0	0	0	6	0	0	0	0	0	0	0	0	0	0	1	0	1
ERR1163807	0	92	0	0	0	0	6	0	0	0	0	0	0	0	0	0	0	1	0	1
ERR1163808	0	88	0	0	0	0	6	0	0	0	0	0	0	0	0	0	0	1	0	1
ERR1163811	0	96	0	0	0	0	6	0	0	0	0	0	0	0	0	0	0	1	0	1
ERR1163814	0	0	91	0	0	6	6	0	8	8	0	0	0	0	0	0	1	0	0	1
ERR1163816	0	76	0	0	0	0	6	0	0	0	0	0	0	0	0	0	0	1	0	1
ERR1163819	0	0	91	0	0	6	6	0	23	8	0	0	0	0	0	0	1	0	0	1
ERR1163834	0	0	91	0	0	6	6	0	23	8	0	0	0	0	0	0	1	0	0	1
ERR1163836	0	0	91	0	0	6	6	0	23	8	0	0	0	0	0	0	1	0	0	1
ERR1163840	0	0	91	0	0	6	0	0	23	8	0	0	0	0	0	0	1	0	0	1
ERR1163845	0	0	9	13	0	6	0	38	15	8	8	0	0	0	1	0	0	0	1	1
ERR1163849	0	100	0	0	0	0	0	0	0	0	0	0	0	0	0	0	0	1	0	1
ERR1163855	0	0	100	0	0	6	0	0	23	8	0	0	0	0	0	0	1	0	0	1
ERR1163861	0	0	91	0	0	6	0	0	23	8	0	0	0	0	0	0	1	0	0	1
ERR1163867	0	0	91	0	0	6	6	0	23	8	0	0	0	0	0	0	1	0	0	1
ERR1163868	0	0	91	0	0	0	0	0	15	8	0	0	0	0	0	0	1	0	0	1
ERR1163869	0	0	91	0	0	6	0	0	23	8	0	0	0	0	0	0	1	0	0	1
ERR1163871	0	0	91	0	0	6	0	0	23	8	0	0	0	0	0	0	1	0	0	1
ERR1163874	0	100	0	0	0	0	6	0	0	0	0	0	0	0	0	0	0	1	0	1
ERR1163883	14	0	0	0	0	0	6	0	0	0	0	0	0	0	1	0	0	0	1	1
ERR1163887	0	0	91	0	0	6	0	38	15	8	0	0	0	0	0	0	1	0	1	1
ERR1512563	0	100	0	0	0	0	6	0	0	0	0	0	0	0	0	0	0	1	0	1
ERR1512588	0	100	0	0	0	0	0	0	0	0	0	0	0	0	0	0	0	1	0	1
ERR1512637	0	100	0	0	0	0	6	0	0	0	0	0	0	0	0	0	0	1	0	1
ERR1512735	0	0	91	0	0	6	6	0	15	8	0	0	0	0	0	0	1	0	0	1
ERR1512777	0	96	0	0	0	0	6	0	0	0	0	0	0	0	0	0	0	1	0	1
ERR1512803	0	0	82	0	0	6	6	0	23	8	0	0	0	0	0	0	1	0	0	1
ERR1512839	0	0	91	0	0	6	6	0	23	8	0	0	0	0	0	0	1	0	0	1
ERR1512845	0	0	100	0	0	6	6	0	23	8	0	0	0	0	0	0	1	0	0	1
ERR1512882	0	92	0	0	0	0	6	0	0	0	0	0	0	0	0	0	0	1	0	1
ERR152091	0	0	100	0	0	6	6	0	23	8	0	0	0	0	0	0	1	0	0	1
ERR152094	0	100	0	0	0	0	0	0	0	0	0	0	0	0	0	0	0	1	0	1
ERR152095	0	100	0	0	0	0	0	0	0	0	0	0	0	0	0	0	0	1	0	1
ERR152097	0	0	100	0	7	6	0	0	23	8	0	0	0	0	0	0	1	0	0	1
ERR152104	0	0	91	0	0	6	100	0	23	8	0	0	0	0	0	0	1	0	0	1
ERR152112	0	0	100	0	7	6	13	0	23	17	0	0	0	0	0	0	1	0	0	1
ERR152120	0	0	100	0	7	6	13	0	23	8	0	0	0	0	0	0	1	0	1	1
ERR152121	0	0	100	0	7	6	19	0	23	17	0	0	0	0	0	0	1	0	0	1
ERR152125	0	0	100	0	7	6	6	0	23	8	0	0	0	0	0	0	1	0	0	1
ERR152126	0	0	100	7	0	12	6	0	23	8	0	0	0	0	0	0	1	0	0	1
ERR152165	0	0	100	0	7	6	6	38	23	17	0	0	0	0	0	0	1	0	1	1
ERR152175	0	0	100	0	0	6	6	0	23	8	0	0	0	0	0	0	1	0	0	1
ERR152314	0	0	100	0	7	6	6	0	23	8	0	0	0	0	0	0	1	0	0	1
ERR152320	0	0	100	0	0	6	0	0	23	8	0	0	0	0	0	0	1	0	0	1
ERR152321	0	0	100	0	7	6	6	0	23	8	0	0	0	0	0	0	1	0	0	1
ERR152323	0	100	0	0	0	0	0	0	0	0	0	0	0	0	0	0	0	1	0	1
ERR152343	0	0	100	0	7	6	13	0	23	8	0	0	0	0	0	0	1	0	0	1
ERR152347	0	0	100	0	0	6	6	0	23	8	0	0	0	0	0	0	1	0	0	1
ERR152349	0	0	100	0	0	6	6	0	23	8	0	0	0	0	0	0	1	0	0	1
ERR152352	0	0	100	0	0	12	0	0	23	8	0	0	0	0	0	0	1	0	0	1
ERR1810311	29	8	9	0	0	0	19	38	0	0	0	0	0	0	1	0	0	0	1	1
ERR1810312	29	0	100	7	7	6	13	0	23	17	0	0	0	0	0	0	1	0	0	1
ERR1810313	0	0	100	0	7	6	6	0	23	17	0	0	0	0	0	0	1	0	0	1
ERR1810314	29	100	0	0	0	0	0	0	0	0	0	0	0	0	0	0	0	1	0	1
ERR1810315	0	0	100	7	0	6	13	0	23	17	0	0	0	0	0	0	1	0	0	1
ERR1810316	14	0	100	7	7	6	0	0	23	17	0	0	0	0	0	0	1	0	0	1
ERR1810317	29	0	100	7	7	6	13	0	23	17	0	0	0	0	0	0	1	0	0	1
ERR1810318	0	0	100	0	0	6	13	0	23	17	0	0	0	0	0	0	1	0	0	1
ERR1810320	29	0	100	0	0	6	19	0	23	17	0	0	0	0	0	0	1	0	0	1
ERR1810325	14	4	100	7	7	6	44	0	23	17	0	0	0	0	0	0	1	0	0	1
ERR1810327	14	100	0	0	0	0	6	0	0	0	0	0	0	0	0	0	0	1	0	1

ERR1851471	29	100	0	0	0	0	0	0	0	0	0	0	0	0	0	0	0	1	0	1
ERR1851473	0	0	100	0	7	6	6	0	23	17	0	0	0	0	0	0	1	0	0	1
ERR1851475	0	0	100	0	0	6	19	0	23	17	0	0	0	0	0	0	1	0	0	1
ERR1884286	0	0	100	7	7	6	13	0	23	17	0	0	0	0	0	0	1	0	0	1
ERR1886541	0	0	100	0	0	6	6	0	23	17	0	0	0	0	0	0	1	0	0	1
ERR1886546	0	0	100	0	0	6	6	0	23	8	0	0	0	0	0	0	1	0	0	1
ERR1886730	0	0	100	7	7	6	19	0	23	17	0	0	0	0	0	0	1	0	0	1
ERR1886731	14	0	100	7	7	6	19	0	23	17	0	0	0	0	0	0	1	0	0	1
ERR1886732	14	0	100	7	7	6	19	0	23	17	0	0	0	0	0	0	1	0	0	1
ERR1886733	0	0	100	7	0	6	13	0	23	17	0	0	0	0	0	0	1	0	0	1
ERR1886734	14	0	100	0	7	6	6	0	23	17	0	0	0	0	0	0	1	0	0	1
ERR1886742	0	0	100	0	7	6	19	0	23	17	0	0	0	0	0	0	1	0	0	1
ERR1886744	14	0	100	7	7	6	13	0	23	17	0	0	0	0	0	0	1	0	0	1
ERR1886745	0	0	100	7	0	6	13	0	23	17	0	0	0	0	0	0	1	0	0	1
ERR1886746	0	0	100	0	7	6	13	0	23	17	0	0	0	0	0	0	1	0	0	1
ERR1886748	0	0	100	13	13	12	13	13	31	25	8	0	0	0	0	0	1	0	0	1
ERR1886786	14	0	100	7	7	6	25	0	23	17	0	0	0	0	0	0	1	0	0	1
ERR553719	0	0	91	0	0	6	0	0	23	8	0	0	0	0	0	0	1	0	0	1
ERR553722	0	0	91	0	7	6	6	0	23	8	0	0	0	0	0	0	1	0	0	1
ERR553725	0	0	91	0	0	6	6	0	23	8	0	0	0	0	0	0	1	0	0	1
ERR553728	0	0	91	0	0	6	6	0	23	8	0	0	0	0	0	0	1	0	0	1
ERR553820	0	100	0	0	0	0	6	0	0	0	0	0	0	0	0	0	0	1	0	1
ERR553822	0	0	91	0	0	6	6	0	23	8	0	0	0	0	0	0	1	0	0	1
ERR553846	0	0	91	0	0	6	6	0	23	8	0	0	0	0	0	0	1	0	0	1
ERR553880	0	92	0	0	0	0	6	0	0	0	0	0	0	0	0	0	0	1	0	1
ERR553884	0	0	91	0	0	0	0	0	15	8	0	0	0	0	0	0	1	0	0	1
ERR593632	0	0	91	0	0	6	0	0	23	8	0	0	0	0	0	0	1	0	0	1
ERR593643	0	0	100	0	0	12	0	0	23	8	0	0	0	0	0	0	1	0	0	1
ERR593662	0	100	0	0	0	0	6	0	0	0	0	0	0	0	0	0	0	1	0	1
ERR593702	0	96	0	0	0	0	6	0	0	0	0	0	0	0	0	0	0	1	0	1
ERR593723	14	0	9	0	0	6	0	38	8	0	0	0	0	0	1	0	0	0	1	1
ERR593728	0	0	91	0	0	6	6	0	15	8	0	0	0	0	0	0	1	0	0	1
ERR593755	14	0	9	0	0	6	0	0	0	0	0	0	0	0	1	0	0	0	1	1
ERR593806	0	0	100	0	0	6	6	0	23	8	0	0	0	0	0	0	1	0	0	1
ERR593807	0	0	100	0	0	6	13	0	23	17	0	0	0	0	0	0	1	0	0	1
ERR593856	0	0	100	0	0	6	0	0	23	17	0	0	0	0	0	0	1	0	0	1
ERR593958	0	0	100	0	0	6	6	0	23	17	0	0	0	0	0	0	1	0	0	1
ERR594029	0	100	0	0	0	0	13	0	0	0	0	0	0	0	0	0	0	1	0	1
ERR757225	0	88	0	0	0	0	0	0	0	0	0	0	0	0	0	0	0	1	0	1
ERR757261	0	84	0	0	0	0	6	0	0	0	0	0	0	0	0	0	0	1	0	1
ERR757263	0	0	100	0	0	6	6	0	15	8	0	0	0	0	0	0	1	0	0	1
ERR757317	0	0	100	0	0	6	6	0	23	8	0	0	0	0	0	0	1	0	0	1
ERR757327	0	0	100	0	0	6	6	0	23	17	0	0	0	0	0	0	1	0	0	1
ERR757334	0	0	100	0	0	6	6	0	23	17	0	0	0	0	0	0	1	0	0	1
ERR757358	0	60	0	0	0	0	0	0	0	0	0	0	0	0	0	0	0	1	0	1
ERR757364	0	80	0	0	0	0	0	0	0	0	0	0	0	0	0	0	0	1	0	1
ERR757370	0	84	0	0	0	0	0	0	0	0	0	0	0	0	0	0	0	1	0	1
ERR757411	0	0	91	0	0	6	6	0	23	8	0	0	0	0	0	0	1	0	0	1
ERR946716	0	84	0	0	0	0	6	0	0	0	0	0	0	0	0	0	0	1	0	1
ERR946719	0	88	0	0	0	0	6	0	0	0	0	0	0	0	0	0	0	1	0	1
ERR946757	0	88	0	0	0	0	0	0	0	0	0	0	0	0	0	0	0	1	0	1
SRR035031	0	0	100	0	0	6	0	0	23	17	0	0	0	0	0	0	1	0	0	1
SRR036813	0	100	0	0	0	0	0	0	0	0	0	0	0	0	0	0	0	1	0	1
SRR036815	0	100	0	0	0	0	6	0	0	0	0	0	0	0	0	0	0	1	0	1
SRR036816	0	100	0	0	0	0	0	0	0	0	0	0	0	0	0	0	0	1	0	1
SRR036817	0	100	0	0	0	0	0	25	0	0	0	0	0	0	0	0	0	1	1	1
SRR036820	0	0	100	0	0	6	0	0	23	17	0	0	0	0	0	0	1	0	0	1
SRR036822	0	0	100	0	0	6	0	0	15	8	0	0	0	0	0	0	1	0	0	1
SRR036837	0	100	0	0	0	0	6	0	0	0	0	0	0	0	0	0	0	1	0	1
SRR036842	0	0	100	0	0	6	0	0	23	17	0	0	0	0	0	0	1	0	0	1
SRR036843	0	0	100	0	7	6	0	0	23	17	0	0	0	0	0	0	1	0	0	1
SRR036844	0	100	0	0	0	0	0	0	0	0	0	0	0	0	0	0	0	1	0	1
SRR036848	0	0	91	0	0	6	6	0	23	17	0	0	0	0	0	0	1	0	0	1
SRR036849	0	100	0	0	0	0	0	25	0	0	0	0	0	0	0	0	0	1	1	1
SRR036852	0	100	0	0	0	0	6	0	0	0	0	0	0	0	0	0	0	1	0	1
SRR1635044	0	0	82	0	0	6	6	0	8	8	0	0	0	0	0	0	1	0	0	1
SRR1635132	0	0	91	0	0	6	6	0	0	8	0	0	0	0	0	0	1	0	0	1
SRR1635135	0	0	82	0	0	0	6	0	8	8	0	0	0	0	0	0	1	0	0	1
SRR1635136	0	0	91	0	0	0	6	0	8	8	0	0	0	0	0	0	1	0	0	1
SRR1635138	0	0	91	0	0	6	6	0	8	8	0	0	0	0	0	0	1	0	0	1
SRR1635147	0	0	91	0	0	0	6	0	8	8	0	0	0	0	0	0	1	0	0	1
SRR1635150	0	0	100	0	0	6	6	0	8	8	0	0	0	0	0	0	1	0	0	1
SRR1635222	0	0	82	0	0	6	0	0	8	8	0	0	0	0	0	0	1	0	0	1
SRR1635236	0	0	82	0	0	0	6	0	0	8	0	0	0	0	0	0	1	0	0	1
SRR1635238	0	0	82	0	0	0	6	0	8	8	0	0	0	0	0	0	1	0	0	1

SRR1635244	0	0	91	0	0	6	6	0	8	8	0	0	0	0	0	0	1	0	0	1
SRR1635259	0	96	0	0	0	0	13	0	0	0	0	0	0	0	0	0	0	1	0	1
SRR1635279	0	0	100	0	0	6	6	0	8	8	0	0	0	0	0	0	1	0	0	1
SRR1714476	0	76	0	0	0	0	0	0	0	0	0	0	0	0	0	0	0	1	0	1
SRR1910452	0	100	0	0	0	0	6	0	0	0	0	0	0	0	0	0	0	1	0	1
SRR1910454	0	96	0	0	0	0	0	0	0	0	0	0	0	0	0	0	0	1	0	1
SRR1910456	0	92	0	0	0	0	6	0	0	0	0	0	0	0	0	0	0	1	0	1
SRR1957307	0	100	0	0	0	0	6	0	0	0	0	0	0	0	0	0	0	1	0	1
SRR1958694	0	100	0	0	0	0	6	0	0	0	0	0	0	0	0	0	0	1	0	1
SRR1975106	14	100	0	0	0	6	0	0	8	0	0	0	0	0	0	0	0	1	0	1
SRR1988398	0	100	0	0	0	0	0	0	0	0	0	0	0	0	0	0	0	1	0	1
SRR1993225	0	100	0	0	0	0	0	0	0	0	0	0	0	0	0	0	0	1	0	1
SRR1993229	0	0	91	0	0	6	0	0	23	8	0	0	0	0	0	0	1	0	0	1
SRR1993233	0	0	91	0	0	6	0	0	23	8	0	0	0	0	0	0	1	0	0	1
SRR2075392	0	84	0	0	0	0	0	0	0	0	0	0	0	0	0	0	0	1	0	1
SRR2075413	0	72	0	0	0	0	0	0	0	0	0	0	0	0	0	0	0	1	0	1
SRR2079614	0	0	100	0	0	6	0	0	15	0	0	0	0	0	0	0	1	0	0	1
SRR2182578	0	68	0	0	0	0	13	0	0	0	0	0	0	0	0	0	0	1	0	1
SRR2182584	0	84	0	0	0	0	6	0	0	0	0	0	0	0	0	0	0	1	0	1
SRR2239872	0	0	0	73	67	24	0	25	31	0	0	0	0	1	0	0	0	0	1	1
SRR2239878	0	0	9	13	20	0	6	25	77	42	0	0	0	1	0	0	0	0	1	1
SRR2423597	0	0	82	7	0	0	0	25	8	8	0	0	0	1	0	0	1	0	1	1
SRR2423598	0	0	82	0	0	6	19	0	15	8	0	0	0	0	0	0	1	0	0	1
SRR2423683	0	0	18	13	33	6	6	25	62	67	8	0	0	1	0	0	0	0	1	1
SRR2423685	0	0	82	7	0	0	0	25	8	8	0	0	0	1	0	0	1	0	1	1
SRR2482990	86	100	0	7	0	6	94	0	8	0	0	0	0	0	0	0	0	1	0	1
SRR2771684	0	84	0	0	0	0	13	0	0	0	0	0	0	0	0	0	0	1	0	1
SRR2771685	0	84	0	0	0	0	0	0	0	0	0	0	0	0	0	0	0	1	0	1
SRR2889309	0	0	18	7	20	6	6	25	77	58	0	0	0	1	0	0	0	0	1	1
SRR2920302	0	0	100	0	0	0	0	0	0	0	0	0	0	0	0	0	1	0	0	1
SRR2920331	0	32	0	0	0	0	0	0	0	0	0	0	0	0	0	0	0	1	0	1
SRR2938817	0	88	0	0	0	0	13	0	0	0	0	0	0	0	0	0	0	1	0	1
SRR2976755	0	0	73	0	0	0	0	25	8	8	0	0	0	1	0	0	1	0	1	1
SRR2976798	0	0	0	47	53	12	0	25	8	17	8	0	0	1	0	0	0	0	1	1
SRR2976802	0	0	91	0	0	6	0	13	0	8	0	0	0	1	0	0	1	0	1	1
SRR2976824	0	0	0	0	13	0	88	25	8	0	0	0	0	1	0	0	0	0	1	1
SRR3066540	0	100	0	0	0	0	6	0	0	0	0	0	0	0	0	0	0	1	0	1
SRR3066541	0	100	0	0	0	0	6	0	0	0	0	0	0	0	0	0	0	1	0	1
SRR3066583	0	84	0	0	0	0	6	0	0	0	0	0	0	0	0	0	0	1	0	1
SRR3066599	0	52	0	0	0	0	0	0	0	0	0	0	0	0	0	0	0	1	0	1
SRR3066602	0	0	27	13	20	0	6	25	77	58	0	0	0	1	0	0	0	0	1	1
SRR3091806	0	36	0	0	0	0	0	0	0	0	0	0	0	0	0	0	0	1	0	1
SRR3091819	0	0	9	7	13	0	0	25	31	67	0	0	0	1	0	0	0	0	1	1
SRR3113802	0	0	73	0	0	0	0	13	8	8	0	0	0	1	0	0	0	0	1	1
SRR3113809	0	0	82	0	0	0	0	13	0	8	0	0	0	1	0	0	1	0	1	1
SRR3113810	0	0	82	7	0	0	0	13	0	8	0	0	0	1	0	0	1	0	1	1
SRR3113822	0	0	100	0	0	0	0	0	0	0	0	0	0	0	0	0	1	0	0	1
SRR3113823	0	44	0	0	0	0	0	0	0	0	0	0	0	0	0	0	0	1	0	1
SRR3113830	0	76	0	0	0	0	6	0	0	0	0	0	0	0	0	0	0	1	0	1
SRR3113835	0	0	0	0	0	0	0	13	31	25	0	0	0	1	0	0	0	0	1	1
SRR3113841	0	60	0	0	0	0	6	0	0	0	0	0	0	0	0	0	0	1	0	1
SRR3113842	0	72	0	0	0	0	0	0	0	0	0	0	0	0	0	0	0	1	0	1
SRR3129706	0	0	100	0	0	6	0	0	8	0	0	0	0	0	0	0	1	0	0	1
SRR3138539	0	0	18	13	13	0	6	13	23	25	0	0	0	1	0	0	0	0	1	1
SRR3138544	0	44	0	0	0	0	0	0	0	0	0	0	0	0	0	0	0	1	0	1
SRR3138552	0	0	9	13	20	0	0	25	23	25	0	0	0	1	0	0	0	0	1	1
SRR3138556	0	32	0	0	0	0	6	0	0	0	0	0	0	0	0	0	0	1	0	1
SRR3138652	0	40	0	0	0	0	0	0	0	0	0	0	0	0	0	0	0	1	0	1
SRR3138656	0	0	27	7	20	0	0	25	62	42	0	0	0	1	0	0	0	0	1	1
SRR3140072	0	0	91	0	0	0	0	0	8	8	0	0	0	0	0	0	1	0	0	1
SRR3140143	0	0	82	7	0	0	0	25	8	8	0	0	0	1	0	0	1	0	1	1
SRR3140145	0	0	91	0	0	0	0	0	0	8	0	0	0	0	0	0	1	0	0	1
SRR3140151	0	0	0	40	33	12	0	25	54	25	0	0	0	1	0	0	0	0	1	1
SRR3140156	0	88	0	0	0	0	13	0	0	0	0	0	0	0	0	0	0	1	0	1
SRR3147874	0	0	0	53	20	71	6	25	15	0	0	0	0	1	0	0	0	0	1	1
SRR3148239	0	0	27	13	27	0	0	25	62	33	0	0	0	1	0	0	0	0	1	1
SRR3171760	100	0	0	0	0	0	0	25	0	0	0	0	0	1	0	0	0	0	1	1
SRR3183452	0	28	0	0	0	0	6	0	0	0	0	0	0	0	0	0	0	1	0	1
SRR3185257	0	0	91	0	0	6	0	25	15	8	0	0	0	1	0	0	1	0	1	1
SRR3185982	0	0	27	20	13	12	6	25	69	42	0	0	0	1	0	0	0	0	1	1
SRR3196131	0	68	0	0	0	0	0	0	0	0	0	0	0	0	0	0	0	1	0	1
SRR3196144	0	0	27	20	27	12	6	25	54	42	0	0	0	1	0	0	0	0	1	1
SRR3196145	0	0	9	13	13	0	0	25	38	67	8	0	0	1	0	0	0	0	1	1
SRR3196684	0	0	82	0	0	0	0	0	0	8	0	0	0	0	0	0	1	0	0	1
SRR3199477	0	0	100	0	0	6	0	0	8	0	0	0	0	0	0	0	1	0	0	1

SRR3201103	0	0	0	27	7	65	0	25	31	0	0	0	0	1	0	0	0	0	1	1
SRR3209765	0	0	9	7	13	0	0	13	62	67	0	0	0	1	0	0	0	0	1	1
SRR3209821	0	80	0	0	0	0	0	13	0	0	0	0	0	1	0	0	0	1	1	1
SRR3214473	43	0	100	7	0	6	6	0	8	8	0	0	0	0	0	0	1	0	0	1
SRR3214475	43	0	100	7	0	6	6	0	8	0	0	0	0	0	0	0	1	0	0	1
SRR3214479	43	0	100	0	0	6	19	0	15	8	0	0	0	0	0	0	1	0	0	1
SRR3214487	43	0	100	7	0	6	13	0	15	8	0	0	0	0	0	0	1	0	0	1
SRR3214488	14	100	0	0	0	0	13	0	0	0	0	0	0	0	0	0	0	1	0	1
SRR3214496	14	100	0	0	0	0	13	0	0	0	0	0	0	0	0	0	0	1	0	1
SRR3215129	0	0	27	7	20	6	6	25	77	58	0	0	0	1	0	0	0	0	1	1
SRR3215130	14	0	0	0	0	0	0	0	0	0	0	0	0	0	1	0	0	0	1	1
SRR3297733	0	0	91	0	0	0	0	0	8	8	0	0	0	0	0	0	1	0	0	1
SRR3297738	0	0	91	0	0	6	0	13	15	8	0	0	0	1	0	0	1	0	1	1
SRR3319972	0	0	0	0	13	6	0	13	77	67	0	0	0	1	0	0	0	0	1	1
SRR3319976	0	0	18	7	20	0	0	13	62	42	0	0	0	1	0	0	0	0	1	1
SRR3319989	0	0	82	7	0	0	0	13	8	8	0	0	0	1	0	0	1	0	1	1
SRR3321634	0	0	0	53	73	12	0	13	8	17	0	0	0	1	0	0	0	0	1	1
SRR3321638	0	0	82	0	0	0	6	0	0	8	0	0	0	0	0	0	1	0	0	1
SRR3321653	0	0	18	7	20	0	0	13	77	50	0	0	0	1	0	0	0	0	1	1
SRR3321706	0	0	91	0	0	6	6	0	8	8	0	0	0	0	0	0	1	0	0	1
SRR3402084	0	0	18	7	13	6	0	13	38	25	0	0	0	1	0	0	0	0	1	1
SRR3402086	0	0	100	0	0	6	0	0	0	0	0	0	0	0	0	0	1	0	0	1
SRR3405953	0	0	73	0	0	0	6	0	8	17	0	0	0	0	0	0	1	0	0	1
SRR3475788	0	0	27	7	20	0	0	25	54	33	0	0	0	1	0	0	0	0	1	1
SRR3475826	0	32	0	0	0	0	0	0	0	0	0	0	0	0	0	0	0	1	0	1
SRR3479931	0	56	0	0	0	0	6	0	0	0	0	0	0	0	0	0	0	1	0	1
SRR3543490	0	0	73	0	0	0	0	13	0	8	0	0	0	1	0	0	1	0	1	1
SRR3543497	0	0	0	7	13	0	0	13	54	75	0	0	0	1	0	0	0	0	1	1
SRR3543500	0	0	82	0	0	0	0	25	8	8	0	0	0	1	0	0	1	0	1	1
SRR3543508	0	80	0	0	0	0	6	0	0	0	0	0	0	0	0	0	0	1	0	1
SRR3545863	0	96	0	0	0	0	13	0	0	0	0	0	0	0	0	0	0	1	0	1
SRR3545869	0	0	18	7	20	0	6	13	38	25	0	0	0	1	0	0	0	0	1	1
SRR3545873	0	0	9	0	20	0	0	13	46	58	0	0	0	1	0	0	0	0	1	1
SRR3545877	0	0	100	0	0	0	0	0	0	0	0	0	0	0	0	0	1	0	0	1
SRR3575115	0	92	0	0	0	0	13	0	0	0	0	0	0	0	0	0	0	1	0	1
SRR3583319	0	0	18	13	27	0	6	13	69	58	0	0	0	1	0	0	0	0	1	1
SRR3583320	0	0	82	0	0	0	0	0	8	8	0	0	0	0	0	0	1	0	0	1
SRR3611569	0	80	9	0	0	0	0	13	0	0	0	0	0	1	0	0	0	1	1	1
SRR3611583	0	92	0	0	0	0	0	0	0	0	0	0	0	0	0	0	0	1	0	1
SRR3611600	0	0	27	13	7	6	0	13	54	25	0	0	0	1	0	0	0	0	1	1
SRR3611601	0	88	0	0	0	0	6	0	0	0	0	0	0	0	0	0	0	1	0	1
SRR3611739	0	0	91	0	7	0	0	0	0	0	0	0	0	0	0	0	1	0	0	1
SRR3611754	0	0	18	7	7	6	0	13	38	33	0	0	0	1	0	0	0	0	1	1
SRR3611756	0	36	0	0	0	0	6	0	0	0	0	0	0	0	0	0	0	1	0	1
SRR3611757	0	0	9	7	0	0	0	0	31	33	0	0	0	1	0	0	0	0	1	1
SRR3611766	0	0	9	7	20	0	0	0	38	42	0	0	0	1	0	0	0	0	1	1
SRR3613717	0	76	0	0	0	0	0	0	0	0	0	0	0	0	0	0	0	1	0	1
SRR3613718	0	92	0	0	0	0	0	0	0	0	0	0	0	0	0	0	0	1	0	1
SRR3658686	29	100	0	0	0	12	0	13	0	0	0	0	0	0	0	0	0	1	0	1
SRR3666682	0	92	0	0	0	0	6	0	0	0	0	0	0	0	0	0	0	1	0	1
SRR3666686	0	0	91	0	0	0	0	0	15	8	0	0	0	0	0	0	1	0	0	1
SRR3666720	0	0	18	20	33	12	6	13	62	42	0	0	0	1	0	0	0	0	1	1
SRR3666722	0	0	100	0	0	0	19	0	8	0	0	0	0	0	0	0	1	0	0	1
SRR3710272	0	40	0	0	0	0	13	0	0	0	0	0	0	0	0	0	0	1	0	1
SRR3726426	0	0	0	80	73	29	0	13	38	17	0	0	0	1	0	0	0	0	1	1
SRR3726442	0	0	82	7	0	0	0	13	0	8	0	0	0	1	0	0	1	0	1	1
SRR3774367	0	0	91	0	0	6	0	13	15	8	0	0	0	1	0	0	1	0	1	1
SRR3824722	0	0	91	0	0	6	0	0	8	0	0	0	0	0	0	0	1	0	0	1
SRR3824724	0	0	100	0	0	6	0	38	15	8	0	0	0	0	0	0	1	0	1	1
SRR3928613	0	96	0	0	0	0	13	0	0	0	0	0	0	0	0	0	0	1	0	1
SRR3947397	0	48	0	0	0	0	6	0	0	0	0	0	0	0	0	0	0	1	0	1
SRR3953099	0	0	91	0	0	6	0	0	0	8	0	0	0	0	0	0	1	0	0	1
SRR3953100	0	0	18	7	13	0	0	13	38	33	0	0	0	1	0	0	0	0	1	1
SRR3953111	0	0	0	60	60	18	0	13	38	17	0	0	0	1	0	0	0	0	1	1
SRR3954068	0	0	9	13	0	0	6	38	0	0	0	0	0	0	1	0	0	0	1	1
SRR3995978	0	0	91	0	0	0	0	0	8	8	0	0	0	0	0	0	1	0	0	1
SRR4005434	0	84	0	0	0	0	0	0	0	0	0	0	0	0	0	0	0	1	0	1
SRR4014383	0	0	100	0	0	6	13	0	15	8	0	0	0	0	0	0	1	0	0	1
SRR4018332	0	0	100	0	0	0	0	0	0	0	0	0	0	0	0	0	1	0	0	1
SRR4035437	0	0	82	0	0	0	0	0	8	8	0	0	0	0	0	0	1	0	0	1
SRR4048185	0	0	91	0	0	6	6	0	8	8	0	0	0	0	0	0	1	0	0	1
SRR4066769	0	0	91	0	0	6	0	38	15	8	0	0	0	0	0	0	1	0	1	1
SRR4066770	0	0	100	0	0	6	0	0	15	8	0	0	0	0	0	0	1	0	0	1
SRR4066781	0	92	0	0	0	0	6	0	0	0	0	0	0	0	0	0	0	1	0	1
SRR4066792	0	100	0	0	0	0	0	0	0	0	0	0	0	0	0	0	0	1	0	1

SRR4066803	0	96	0	0	0	0	0	0	0	0	0	0	0	0	0	0	0	1	0	1
SRR4066838	0	96	0	0	0	0	6	0	0	0	0	0	0	0	0	0	0	1	0	1
SRR4066859	14	100	0	0	0	0	19	0	0	0	0	0	0	0	0	0	0	1	0	1
SRR4066861	0	0	100	0	0	6	0	0	8	0	0	0	0	0	0	0	1	0	0	1
SRR4066911	0	0	100	7	0	0	0	0	8	8	0	0	0	0	0	0	1	0	0	1
SRR4066929	0	100	0	0	0	0	6	0	0	0	0	0	0	0	0	0	0	1	0	1
SRR4067166	0	100	0	0	0	0	6	0	0	0	0	0	0	0	0	0	0	1	0	1
SRR4125004	0	0	82	7	0	0	0	13	0	8	0	0	0	1	0	0	1	0	1	1
SRR4125006	0	88	0	0	0	0	6	0	0	0	0	0	0	0	0	0	0	1	0	1
SRR4125011	0	80	0	0	0	0	0	0	0	0	0	0	0	0	0	0	0	1	0	1
SRR4175493	0	84	0	0	0	0	0	0	0	0	0	0	0	0	0	0	0	1	0	1
SRR4175495	0	0	9	13	20	12	6	13	54	42	0	0	0	1	0	0	0	0	1	1
SRR4204497	0	0	82	0	0	6	0	0	0	8	0	0	0	0	0	0	1	0	0	1
SRR4242483	0	96	0	0	0	0	6	0	0	0	0	0	0	0	0	0	0	1	0	1
SRR4242494	0	0	91	0	0	6	0	0	0	8	0	0	0	0	0	0	1	0	0	1
SRR4242496	0	96	0	0	0	0	6	0	0	0	0	0	0	0	0	0	0	1	0	1
SRR4242504	14	0	0	0	0	0	0	38	0	0	0	0	0	0	1	0	0	0	1	1
SRR4242506	0	100	0	0	0	0	19	0	0	0	0	0	0	0	0	0	0	1	0	1
SRR4242518	0	96	0	0	0	0	6	0	0	0	0	0	0	0	0	0	0	1	0	1
SRR4280308	0	0	100	0	0	6	0	0	8	0	0	0	0	0	0	0	1	0	0	1
SRR4280309	0	80	0	0	0	0	0	0	0	0	0	0	0	0	0	0	0	1	0	1
SRR4281437	0	72	0	0	0	0	6	0	0	0	0	0	0	0	0	0	0	1	0	1
SRR4281462	0	68	0	0	0	0	0	0	0	0	0	0	0	0	0	0	0	1	0	1
SRR4281678	0	68	0	0	0	0	0	0	0	0	0	0	0	0	0	0	0	1	0	1
SRR4283841	0	88	0	0	0	0	6	0	0	0	0	0	0	0	0	0	0	1	0	1
SRR4283854	0	48	0	0	0	0	0	0	0	0	0	0	0	0	0	0	0	1	0	1
SRR4288976	0	0	91	0	0	6	19	0	15	8	0	0	0	0	0	0	1	0	0	1
SRR4301049	0	0	91	0	0	6	0	0	8	0	0	0	0	0	0	0	1	0	0	1
SRR4301050	0	0	100	0	0	6	0	0	8	0	0	0	0	0	0	0	1	0	0	1
SRR4301051	0	80	0	0	0	0	6	0	0	0	0	0	0	0	0	0	0	1	0	1
SRR4301071	0	0	100	0	0	0	6	0	0	0	0	0	0	0	0	0	1	0	0	1
SRR4301088	0	92	0	0	0	0	6	0	0	0	0	0	0	0	0	0	0	1	0	1
SRR4301153	0	72	0	0	0	0	6	0	0	0	0	0	0	0	0	0	0	1	0	1
SRR4301243	0	0	9	7	13	0	0	13	46	33	0	0	0	1	0	0	0	0	1	1
SRR4301253	0	84	0	0	0	0	0	0	0	0	0	0	0	0	0	0	0	1	0	1
SRR4340550	14	100	9	0	0	0	6	0	0	0	0	0	0	0	0	0	0	1	0	1
SRR4342163	0	88	0	0	0	0	13	0	0	0	0	0	0	0	0	0	0	1	0	1
SRR4412626	0	0	100	0	0	0	0	0	0	0	0	0	0	0	0	0	1	0	0	1
SRR4434188	0	92	0	0	0	0	6	0	0	0	0	0	0	0	0	0	0	1	0	1
SRR4434189	14	0	0	0	0	0	6	0	0	0	0	0	0	0	1	0	0	0	1	1
SRR4434195	0	0	82	0	0	0	0	0	0	0	0	0	0	0	0	0	1	0	0	1
SRR4434207	0	92	0	0	0	0	0	0	0	0	0	0	0	0	0	0	0	1	0	1
SRR4453631	14	0	0	0	0	0	6	0	0	0	0	0	0	0	1	0	0	0	1	1
SRR4453632	0	80	0	0	0	0	6	0	0	0	0	0	0	0	0	0	0	1	0	1
SRR4453634	0	92	0	0	0	0	6	0	0	0	0	0	0	0	0	0	0	1	0	1
SRR4453641	0	0	100	0	0	6	0	0	8	0	0	0	0	0	0	0	1	0	0	1
SRR4453656	0	88	0	0	0	0	0	0	0	0	0	0	0	0	0	0	0	1	0	1
SRR4453661	0	60	0	0	0	0	0	0	0	0	0	0	0	0	0	0	0	1	0	1
SRR4453669	0	0	18	13	13	0	0	13	38	33	0	0	0	1	0	0	0	0	1	1
SRR4453677	0	88	0	0	0	0	6	0	0	0	0	0	0	0	0	0	0	1	0	1
SRR4453680	0	72	0	0	0	0	6	0	0	0	0	0	0	0	0	0	0	1	0	1
SRR4453727	0	68	0	0	0	0	6	0	0	0	0	0	0	0	0	0	0	1	0	1
SRR5005383	0	0	82	0	0	6	0	0	15	17	0	0	0	0	0	0	1	0	0	1
SRR5005386	0	0	82	7	0	0	0	13	8	8	0	0	0	1	0	0	1	0	1	1
SRR5005390	0	0	91	0	0	6	0	0	23	8	0	0	0	0	0	0	1	0	0	1
SRR5005393	0	0	100	0	0	6	6	0	8	0	0	0	0	0	0	0	1	0	0	1
SRR5019876	0	88	0	0	0	0	6	0	0	0	0	0	0	0	0	0	0	1	0	1
SRR5019877	0	0	91	0	0	0	6	0	0	8	0	0	0	0	0	0	1	0	0	1
SRR5019878	0	80	0	0	0	0	0	0	0	0	0	0	0	0	0	0	0	1	0	1
SRR5019881	0	0	73	0	0	0	6	0	0	8	0	0	0	0	0	0	1	0	0	1
SRR5043954	0	0	100	0	0	0	0	0	0	0	0	0	0	0	0	0	1	0	0	1
SRR5045349	0	0	9	13	20	6	0	13	62	33	0	0	0	1	0	0	0	0	1	1
SRR5045350	0	92	0	0	0	0	6	0	0	0	0	0	0	0	0	0	0	1	0	1
SRR5045358	0	0	82	7	0	0	0	13	8	8	0	0	0	1	0	0	1	0	1	1
SRR5045362	0	0	9	40	33	65	0	13	8	0	0	0	0	1	0	0	0	0	1	1
SRR5047591	0	84	0	0	0	0	0	0	0	0	0	0	0	0	0	0	0	1	0	1
SRR5047599	0	0	0	0	13	0	88	13	8	0	0	0	0	1	0	0	0	0	1	1
SRR5079930	0	96	0	0	0	0	6	0	0	0	0	0	0	0	0	0	0	1	0	1
SRR5079931	0	96	0	0	0	0	6	0	0	0	0	0	0	0	0	0	0	1	0	1
SRR5079935	0	84	0	0	0	0	0	0	0	0	0	0	0	0	0	0	0	1	0	1
SRR5080887	0	80	0	0	0	0	19	0	0	0	0	0	0	0	0	0	0	1	0	1
SRR5080902	0	0	91	0	0	0	6	0	0	0	0	0	0	0	0	0	1	0	0	1
SRR5080903	0	0	100	0	0	0	0	0	8	0	0	0	0	0	0	0	1	0	0	1
SRR5091751	0	76	0	0	0	0	0	0	0	0	0	0	0	0	0	0	0	1	0	1
SRR5091752	0	76	0	0	0	0	0	0	0	0	0	0	0	0	0	0	0	1	0	1

SRR5091762	0	60	0	0	0	0	0	0	0	0	0	0	0	0	0	0	0	1	0	1
SRR5091856	0	0	100	0	0	0	0	0	0	0	0	0	0	0	0	0	1	0	0	1
SRR5091859	0	0	100	0	0	6	6	0	8	0	0	0	0	0	0	0	1	0	0	1
SRR5091865	0	0	100	0	0	0	0	0	8	0	0	0	0	0	0	0	1	0	0	1
SRR5091868	0	0	82	0	0	6	0	0	8	8	0	0	0	0	0	0	1	0	0	1
SRR5091874	0	88	0	0	0	0	13	0	0	0	0	0	0	0	0	0	0	1	0	1
SRR5106055	0	84	0	0	0	0	0	0	0	0	0	0	0	0	0	0	0	1	0	1
SRR5106057	0	84	0	0	0	0	0	0	0	0	0	0	0	0	0	0	0	1	0	1
SRR5106162	0	0	91	0	0	12	0	0	15	8	0	0	0	0	0	0	1	0	0	1
SRR5119517	0	0	82	0	0	0	0	0	0	0	0	0	0	0	0	0	1	0	0	1
SRR5121010	0	0	9	20	13	18	0	13	69	50	0	0	0	1	0	0	0	0	1	1
SRR5121017	0	92	0	0	0	0	13	0	0	0	0	0	0	0	0	0	0	1	0	1
SRR5121020	0	0	0	0	13	0	88	13	8	0	0	0	0	1	0	0	0	0	1	1
SRR5127262	0	96	0	0	0	0	13	0	0	0	0	0	0	0	0	0	0	1	0	1
SRR5129694	0	60	0	0	0	0	6	0	0	0	0	0	0	0	0	0	0	1	0	1
SRR5129727	0	0	0	20	47	12	0	13	23	17	0	0	0	1	0	0	0	0	1	1
SRR5129921	0	0	9	7	13	0	0	0	62	33	0	0	0	1	0	0	0	0	1	1
SRR5129932	0	56	0	0	0	0	6	0	0	0	0	0	0	0	0	0	0	1	0	1
SRR5132302	0	84	0	0	0	0	0	0	0	0	0	0	0	0	0	0	0	1	0	1
SRR5137103	0	88	0	0	0	0	0	0	0	0	0	0	0	0	0	0	0	1	0	1
SRR5137106	0	0	91	0	0	6	0	0	0	17	0	0	0	0	0	0	1	0	0	1
SRR5139820	0	88	0	0	0	0	13	0	0	0	0	0	0	0	0	0	0	1	0	1
SRR5139841	86	0	0	0	0	6	0	13	0	0	0	0	0	1	0	0	0	0	1	1
SRR5139842	0	0	0	20	7	47	0	25	8	0	0	0	0	1	0	0	0	0	1	1
SRR5139844	0	68	0	0	0	0	0	0	0	0	0	0	0	0	0	0	0	1	0	1
SRR5139863	0	0	82	0	0	0	19	13	0	8	0	0	0	1	0	0	1	0	1	1
SRR5152188	0	0	0	0	7	0	0	13	38	58	0	0	0	1	0	0	0	0	1	1
SRR5152194	0	0	82	0	0	0	0	13	0	8	0	0	0	1	0	0	1	0	1	1
SRR5152206	0	0	27	13	20	6	0	13	46	42	0	0	0	1	0	0	0	0	1	1
SRR5152208	0	100	0	0	0	0	6	0	0	0	0	0	0	0	0	0	0	1	0	1
SRR5152212	0	60	0	0	0	0	6	0	0	0	0	0	0	0	0	0	0	1	0	1
SRR5152311	0	96	0	0	0	0	13	0	0	0	0	0	0	0	0	0	0	1	0	1
SRR5152313	14	8	0	0	0	0	6	13	0	0	0	0	1	0	0	0	0	0	1	1
SRR5152316	0	0	100	0	0	6	0	0	8	0	0	0	0	0	0	0	1	0	0	1
SRR5164540	0	0	100	0	0	0	19	0	0	0	0	0	0	0	0	0	1	0	0	1
SRR5170909	0	0	0	60	67	18	0	13	38	17	0	0	0	1	0	0	0	0	1	1
SRR5200968	0	0	100	0	0	6	0	0	8	0	0	0	0	0	0	0	1	0	0	1
SRR5209859	0	60	0	0	0	0	0	0	0	0	0	0	0	0	0	0	0	1	0	1
SRR5209897	0	0	82	7	0	0	0	13	8	8	0	0	0	1	0	0	1	0	1	1
SRR5209920	0	44	0	0	0	0	0	0	0	0	0	0	0	0	0	0	0	1	0	1
SRR5222007	0	96	0	0	0	0	19	0	0	0	0	0	0	0	0	0	0	1	0	1
SRR5230143	0	56	0	0	0	0	0	38	0	0	0	0	0	0	0	0	0	1	1	1
SRR5230167	0	92	0	0	0	0	0	0	0	0	0	0	0	0	0	0	0	1	0	1
SRR5251091	0	8	82	0	0	0	13	0	0	8	0	0	0	0	0	0	1	0	0	1
SRR5251173	0	72	0	0	0	0	0	0	0	0	0	0	0	0	0	0	0	1	0	1
SRR5251186	0	0	100	0	0	6	6	0	8	0	0	0	0	0	0	0	1	0	0	1
SRR5251600	0	72	0	0	0	0	6	0	0	0	0	0	0	0	0	0	0	1	0	1
SRR5251621	0	80	0	0	0	0	13	0	0	0	0	0	0	0	0	0	0	1	0	1
SRR5271437	0	44	0	0	0	0	13	0	0	0	0	0	0	0	0	0	0	1	0	1
SRR5271495	0	44	0	0	0	0	13	0	0	0	0	0	0	0	0	0	0	1	0	1
SRR5272716	0	0	100	0	0	0	19	0	0	0	0	0	0	0	0	0	1	0	0	1
SRR5278114	0	76	0	0	0	0	0	0	0	0	0	0	0	0	0	0	0	1	0	1
SRR5278123	0	64	0	7	0	0	6	0	0	0	0	0	0	0	0	0	0	1	0	1
SRR5297340	0	0	9	53	47	18	0	25	46	33	0	0	0	1	0	0	0	0	1	1
SRR5297365	0	0	27	13	27	12	6	13	77	50	0	0	0	1	0	0	0	0	1	1
SRR5336387	0	0	18	20	27	0	6	13	69	42	0	0	0	1	0	0	0	0	1	1
SRR5336548	0	0	100	0	0	0	6	0	0	8	0	0	0	0	0	0	1	0	0	1
SRR5336551	0	92	0	0	0	0	0	0	0	0	0	0	0	0	0	0	0	1	0	1
SRR5336562	0	0	91	0	0	6	0	0	15	8	0	0	0	0	0	0	1	0	0	1
SRR5336608	0	0	18	27	20	0	6	25	69	58	0	0	0	1	0	0	0	0	1	1
SRR5344911	0	64	0	0	0	0	6	0	0	0	0	0	0	0	0	0	0	1	0	1
SRR5344977	14	0	0	0	0	0	0	0	0	0	0	0	0	0	1	0	0	0	1	1
SRR5345314	0	76	0	0	0	0	0	0	0	0	0	0	0	0	0	0	0	1	0	1
SRR5345358	0	96	0	0	0	0	13	0	0	0	0	0	0	0	0	0	0	1	0	1
SRR5345425	0	84	0	0	0	0	0	0	0	0	0	0	0	0	0	0	0	1	0	1
SRR5345431	0	84	0	0	0	0	13	0	0	0	0	0	0	0	0	0	0	1	0	1
SRR5345447	0	52	0	0	0	0	0	0	0	0	0	0	0	0	0	0	0	1	0	1
SRR5351013	0	80	0	0	0	0	0	0	0	0	0	0	0	0	0	0	0	1	0	1
SRR5414327	0	60	0	0	0	0	0	0	0	0	0	0	0	0	0	0	0	1	0	1
SRR5414334	0	68	0	0	0	0	0	0	0	0	0	0	0	0	0	0	0	1	0	1
SRR5414348	0	68	0	0	0	0	0	0	0	0	0	0	0	0	0	0	0	1	0	1
SRR5414358	0	80	0	0	0	0	0	0	0	0	0	0	0	0	0	0	0	1	0	1
SRR5414369	0	76	0	0	0	0	0	0	0	0	0	0	0	0	0	0	0	1	0	1
SRR5414372	0	84	0	0	0	0	6	0	0	0	0	0	0	0	0	0	0	1	0	1
SRR5414373	0	76	0	0	0	0	6	0	0	0	0	0	0	0	0	0	0	1	0	1

SRR5414378	0	72	0	0	0	0	0	0	0	0	0	0	0	0	0	0	0	1	0	1
SRR5414382	0	92	0	0	0	0	13	0	0	0	0	0	0	0	0	0	0	1	0	1
SRR5414389	0	0	100	0	0	0	0	0	0	0	0	0	0	0	0	0	1	0	0	1
SRR5414390	0	0	91	0	0	0	0	0	0	0	0	0	0	0	0	0	1	0	0	1
SRR5414395	0	88	0	0	0	0	13	0	0	0	0	0	0	0	0	0	0	1	0	1
SRR5414404	0	76	0	0	0	0	13	0	0	0	0	0	0	0	0	0	0	1	0	1
SRR5414419	0	64	0	0	0	0	0	0	0	0	0	0	0	0	0	0	0	1	0	1
SRR5414433	0	84	0	0	0	0	6	0	0	0	0	0	0	0	0	0	0	1	0	1
SRR5414434	0	68	0	0	0	0	0	0	0	0	0	0	0	0	0	0	0	1	0	1
SRR5414435	0	68	0	0	0	0	0	0	0	0	0	0	0	0	0	0	0	1	0	1
SRR5414439	0	80	0	0	0	0	0	0	0	0	0	0	0	0	0	0	0	1	0	1
SRR5414441	0	92	0	0	0	0	0	0	0	0	0	0	0	0	0	0	0	1	0	1
SRR5414447	0	0	100	0	0	0	0	0	0	0	0	0	0	0	0	0	1	0	0	1
SRR5414455	0	0	73	7	0	0	6	0	0	0	0	0	0	0	0	0	1	0	0	1
SRR5414456	14	0	0	0	0	6	0	0	0	0	0	0	0	0	1	0	0	0	1	1
SRR5414458	0	60	0	0	0	0	0	0	0	0	0	0	0	0	0	0	0	1	0	1
SRR5414462	0	44	0	0	0	0	6	0	0	0	0	0	0	0	0	0	0	1	0	1
SRR5414478	0	0	91	0	0	6	0	0	0	8	0	0	0	0	0	0	1	0	0	1
SRR5414479	0	60	0	0	0	0	6	0	0	0	0	0	0	0	0	0	0	1	0	1
SRR5414494	0	40	0	0	0	0	6	0	0	0	0	0	0	0	0	0	0	1	0	1
SRR5414502	14	0	0	0	0	0	0	0	0	0	0	0	0	0	1	0	0	0	1	1
SRR5414508	0	0	91	0	0	0	6	0	8	0	0	0	0	0	0	0	1	0	0	1
SRR5414523	0	0	100	0	0	0	0	0	0	0	0	0	0	0	0	0	1	0	0	1
SRR5414534	0	0	91	0	0	0	13	0	0	0	0	0	0	0	0	0	1	0	0	1
SRR5414538	0	56	0	0	0	0	0	0	0	0	0	0	0	0	0	0	0	1	0	1
SRR5414539	0	28	0	0	0	6	0	0	0	0	0	0	0	0	0	0	0	1	0	1
SRR5414550	0	48	0	0	0	0	13	0	0	0	0	0	0	0	0	0	0	1	0	1
SRR5414552	0	80	0	0	0	0	0	0	0	0	0	0	0	0	0	0	0	1	0	1
SRR5414556	0	80	0	0	0	0	0	0	0	0	0	0	0	0	0	0	0	1	0	1
SRR5414574	0	64	0	0	0	0	0	0	0	0	0	0	0	0	0	0	0	1	0	1
SRR5414580	0	64	0	0	0	0	13	0	0	0	0	0	0	0	0	0	0	1	0	1
SRR5414582	0	0	91	0	0	0	0	0	0	0	0	0	0	0	0	0	1	0	0	1
SRR5414583	0	0	91	0	0	0	0	0	0	0	0	0	0	0	0	0	1	0	0	1
SRR5414587	0	0	82	0	0	0	0	0	0	0	0	0	0	0	0	0	1	0	0	1
SRR5414588	0	0	100	0	0	0	0	0	0	0	0	0	0	0	0	0	1	0	0	1
SRR5414595	0	0	91	0	0	0	0	0	0	0	0	0	0	0	0	0	1	0	0	1
SRR5414601	0	60	0	0	0	0	0	0	0	0	0	0	0	0	0	0	0	1	0	1
SRR5414602	0	60	0	0	0	0	0	0	0	0	0	0	0	0	0	0	0	1	0	1
SRR5414608	0	60	0	0	0	0	0	0	0	0	0	0	0	0	0	0	0	1	0	1
SRR5414610	0	36	0	0	0	0	0	0	0	0	0	0	0	0	0	0	0	1	0	1
SRR5414614	0	48	0	0	0	0	0	0	0	0	0	0	0	0	0	0	0	1	0	1
SRR5414618	0	0	73	0	0	0	0	0	0	0	0	0	0	0	0	0	1	0	0	1
SRR5414619	0	72	0	0	0	0	0	0	0	0	0	0	0	0	0	0	0	1	0	1
SRR5414624	0	40	0	0	0	0	0	0	0	0	0	0	0	0	0	0	0	1	0	1
SRR5414625	0	0	100	0	0	0	0	0	0	0	0	0	0	0	0	0	1	0	0	1
SRR5414626	0	48	0	0	0	0	13	0	0	0	0	0	0	0	0	0	0	1	0	1
SRR5414648	0	68	0	0	0	0	6	0	0	0	0	0	0	0	0	0	0	1	0	1
SRR5414650	0	56	0	0	0	0	6	0	0	0	0	0	0	0	0	0	0	1	0	1
SRR5414665	0	80	0	0	0	0	13	0	0	0	0	0	0	0	0	0	0	1	0	1
SRR5414670	0	0	91	0	0	0	19	0	0	0	0	0	0	0	0	0	1	0	0	1
SRR5414675	0	60	0	0	0	0	0	0	0	0	0	0	0	0	0	0	0	1	0	1
SRR5414681	0	68	0	0	0	0	13	0	0	0	0	0	0	0	0	0	0	1	0	1
SRR5414685	0	52	0	0	0	0	13	0	0	0	0	0	0	0	0	0	0	1	0	1
SRR5414700	0	28	0	0	0	0	0	0	0	0	0	0	0	0	0	0	0	1	0	1
SRR5414701	0	92	0	0	0	0	0	0	0	0	0	0	0	0	0	0	0	1	0	1
SRR5414728	0	0	91	0	0	0	0	0	0	0	0	0	0	0	0	0	1	0	0	1
SRR5414751	0	72	0	0	0	0	0	0	0	0	0	0	0	0	0	0	0	1	0	1
SRR5414763	0	0	100	0	0	0	0	0	0	0	0	0	0	0	0	0	1	0	0	1
SRR5414765	0	0	73	0	0	0	19	0	0	0	0	0	0	0	0	0	1	0	0	1
SRR5414769	0	56	0	0	0	0	0	0	0	0	0	0	0	0	0	0	0	1	0	1
SRR5414778	0	24	0	0	0	0	0	0	0	0	0	0	0	0	0	0	0	1	0	1
SRR5414785	0	72	0	0	0	0	13	0	0	0	0	0	0	0	0	0	0	1	0	1
SRR5414793	0	24	0	0	0	0	0	0	0	0	0	0	0	0	0	0	0	1	0	1
SRR5414803	0	64	0	0	0	0	0	0	0	0	0	0	0	0	0	0	0	1	0	1
SRR5414826	0	28	0	0	0	0	0	0	0	0	0	0	0	0	0	0	0	1	0	1
SRR5414837	0	64	0	0	0	0	0	0	0	0	0	0	0	0	0	0	0	1	0	1
SRR5414842	0	52	0	0	0	0	0	0	0	0	0	0	0	0	0	0	0	1	0	1
SRR5414844	0	76	0	0	0	0	13	0	0	0	0	0	0	0	0	0	0	1	0	1
SRR5414846	0	0	100	0	0	0	0	0	0	0	0	0	0	0	0	0	1	0	0	1
SRR5414860	0	80	0	0	0	0	0	0	0	0	0	0	0	0	0	0	0	1	0	1
SRR5414861	0	76	0	0	0	0	0	0	0	0	0	0	0	0	0	0	0	1	0	1
SRR5414863	0	64	0	0	0	0	0	0	0	0	0	0	0	0	0	0	0	1	0	1
SRR5414867	0	80	0	0	0	0	0	0	0	0	0	0	0	0	0	0	0	1	0	1
SRR5414877	0	0	100	0	0	0	0	0	0	0	0	0	0	0	0	0	1	0	0	1
SRR5660090	0	88	0	0	0	0	13	0	0	0	0	0	0	0	0	0	0	1	0	1

SRR5660093	0	0	100	0	0	0	6	0	0	0	0	0	0	0	0	0	1	0	0	1
SRR5660125	14	0	0	0	0	0	0	0	0	0	0	0	0	0	1	0	0	0	1	1
SRR5660147	0	84	0	0	0	0	0	0	0	0	0	0	0	0	0	0	0	1	0	1
SRR5711496	0	0	100	0	0	0	6	0	8	0	0	0	0	0	0	0	1	0	0	1
SRR5712096	0	96	0	0	0	0	6	0	0	0	0	0	0	0	0	0	0	1	0	1
SRR5712097	0	20	0	27	33	0	6	0	15	25	8	0	0	0	0	0	0	1	0	1
SRR5712108	0	96	0	0	0	0	6	0	0	0	0	0	0	0	0	0	0	1	0	1
SRR5794756	14	8	0	0	0	6	0	0	0	0	0	0	0	0	1	0	0	0	1	1
SRR5808800	0	80	0	0	0	0	6	0	0	0	0	0	0	0	0	0	0	1	0	1
SRR5851310	0	0	9	20	13	18	6	25	62	75	0	0	0	1	0	0	0	0	1	1
SRR5851335	0	48	0	0	0	0	0	0	0	0	0	0	0	0	0	0	0	1	0	1
SRR5858141	0	0	27	13	20	12	6	25	46	42	0	0	0	1	0	0	0	0	1	1
SRR5862688	0	64	0	0	0	0	13	0	0	0	0	0	0	0	0	0	0	1	0	1
SRR5862689	0	68	0	0	0	0	13	0	0	0	0	0	0	0	0	0	0	1	0	1
SRR5862691	0	80	0	0	0	0	13	0	0	0	0	0	0	0	0	0	0	1	0	1
SRR5862696	14	0	0	0	0	0	0	0	0	0	0	0	0	0	1	0	0	0	1	1
SRR5862701	0	60	0	0	0	0	6	0	0	0	0	0	0	0	0	0	0	1	0	1
SRR5862987	0	52	0	0	0	0	0	0	0	0	0	0	0	0	0	0	0	1	0	1
SRR5865306	0	0	18	20	13	0	0	13	38	67	0	0	0	1	0	0	0	0	1	1
SRR5865307	0	88	0	0	0	0	6	0	0	0	0	0	0	0	0	0	0	1	0	1
SRR5865311	0	44	0	0	0	0	6	0	0	0	0	0	0	0	0	0	0	1	0	1
SRR5865313	0	64	0	0	0	0	6	0	0	0	0	0	0	0	0	0	0	1	0	1
SRR5865318	0	0	18	7	13	0	6	25	46	42	0	0	0	1	0	0	0	0	1	1
SRR5865319	0	0	9	7	20	0	0	25	69	42	0	0	0	1	0	0	0	0	1	1
SRR5865323	0	0	9	7	13	0	6	25	46	33	0	0	0	1	0	0	0	0	1	1
SRR5865326	0	0	18	7	20	0	6	25	69	42	0	0	0	1	0	0	0	0	1	1
SRR5865327	0	0	18	13	20	0	6	25	62	50	0	0	0	1	0	0	0	0	1	1
ERR1163614	0	0	36	13	7	12	19	50	31	8	8	0	1	0	0	0	0	0	1	2
ERR1163713	0	0	18	7	7	12	6	38	31	25	8	0	1	0	0	0	0	0	1	2
ERR1163416	0	0	0	0	0	0	0	88	0	0	15	0	0	0	0	1	0	0	1	3
ERR1163434	0	0	9	0	13	6	6	38	8	17	8	0	0	0	1	0	0	0	1	3
ERR1163441	0	0	0	0	0	6	0	75	0	0	23	0	0	0	0	1	0	0	1	3
ERR1163494	0	0	0	0	0	0	6	75	0	0	15	0	0	0	0	1	0	0	1	3
ERR1163599	0	0	9	0	7	0	0	88	8	0	23	0	0	0	0	1	0	0	1	3
ERR1163615	0	0	0	0	0	0	0	88	0	0	0	0	0	0	0	1	0	0	1	3
ERR1163639	0	0	0	0	0	0	0	88	0	0	0	0	0	0	0	1	0	0	1	3
ERR1163655	0	0	0	0	0	0	0	100	0	0	8	1	0	0	0	1	0	0	1	3
ERR1163676	0	0	0	0	0	0	0	88	0	0	15	0	0	0	0	1	0	0	1	3
ERR1163708	0	12	9	0	0	0	13	0	0	0	8	0	0	0	0	0	0	1	0	3
ERR1163711	0	8	55	13	13	12	0	50	0	8	8	1	0	0	0	0	1	0	1	3
ERR1163712	0	0	0	7	0	6	6	50	8	0	23	1	0	0	0	0	0	0	1	3
ERR1163717	0	0	0	0	0	0	0	75	0	0	23	0	0	0	0	1	0	0	1	3
ERR1163725	0	0	9	0	0	0	6	88	0	0	15	0	0	0	0	1	0	1	1	3
ERR1163726	0	20	0	13	13	6	6	50	0	0	0	0	0	0	0	0	0	1	1	3
ERR1163731	0	24	0	7	7	6	13	13	0	8	8	0	0	0	0	0	0	1	0	3
ERR1163754	0	12	0	0	7	6	6	50	0	0	8	1	0	0	0	0	0	1	1	3
ERR1163785	0	28	0	53	53	35	6	13	15	17	23	0	0	0	0	0	0	1	0	3
ERR1163786	0	0	0	0	0	0	0	50	0	0	23	1	0	0	0	0	0	0	1	3
ERR1163788	0	28	0	7	7	6	6	13	0	0	0	0	0	0	0	0	0	1	0	3
ERR1163801	0	0	0	0	0	6	0	50	0	8	62	1	0	0	0	0	0	0	1	3
ERR1163806	0	0	45	13	13	12	0	38	0	8	0	1	0	0	0	0	1	0	1	3
ERR1163812	0	0	45	13	13	12	0	0	0	8	8	0	0	0	0	0	1	0	0	3
ERR1163818	0	8	0	0	7	0	6	0	0	0	8	0	0	0	0	0	0	1	0	3
ERR1163826	0	0	0	0	0	0	6	50	8	0	23	1	0	0	0	0	0	0	1	3
ERR1163830	0	8	55	13	13	12	0	0	0	17	8	0	0	0	0	0	1	0	0	3
ERR1163831	0	0	9	0	0	0	0	75	0	8	23	0	0	0	0	1	0	0	1	3
ERR1163833	0	0	9	0	0	0	6	50	0	8	0	0	0	0	1	0	0	0	1	3
ERR1163835	0	16	0	7	7	6	6	13	0	0	0	0	0	0	0	0	0	1	0	3
ERR1163841	0	0	0	0	0	0	0	63	0	0	15	0	0	0	0	1	0	0	1	3
ERR1163854	0	8	45	13	13	12	6	0	0	8	0	0	0	0	0	0	1	0	0	3
ERR1163863	0	0	0	0	0	0	0	75	0	0	8	0	0	0	0	1	0	0	1	3
ERR1163885	0	0	0	0	0	0	0	88	0	0	0	0	0	0	0	1	0	0	1	3
ERR553818	14	0	0	0	0	0	6	13	0	0	0	1	0	0	0	0	0	0	0	3
ERR757232	0	0	9	0	0	0	6	50	0	0	8	0	0	0	1	0	0	0	1	3
SRR1975100	0	0	9	0	0	0	0	88	0	8	23	0	0	0	1	1	0	0	1	3
SRR1975124	0	52	9	20	13	12	6	13	0	8	8	0	0	0	0	0	0	1	0	3
SRR1975129	0	20	9	13	7	18	6	50	8	8	8	1	0	0	0	0	0	1	1	3
SRR2920321	0	0	9	0	0	0	6	50	0	0	0	0	0	0	1	0	0	0	1	3
SRR3214482	14	28	9	27	27	18	25	13	8	0	8	0	0	0	0	0	0	1	0	3
SRR3214492	14	60	0	13	20	12	6	13	0	0	0	0	0	0	0	0	0	1	0	3
SRR5278289	0	32	0	20	13	12	19	0	0	0	8	0	0	0	0	0	0	1	0	3

Supplementary Table 5

Group 2					
**Gene**	**Non-unique Gene name**	**Annotation**	**Orthologue**	**coverage**	**%nt id to *C. jejuni***
**ERR1163614**	**XIII**				
group_7984	pglH_2	GalNAc-alpha-(1->4)-GalNAc-alpha-(1->3)-diNAcBac-PP-undecapreno	4055	50	76
gspA		General stress protein A	4003	N/A	N/A
group_7985	epsH	Putative glycosyltransferase EpsH	4056	36	82
group_7986	cstIII	GT-42 glycosyltransferase	4057	100	82
legI_1		N,N'-diacetyllegionaminic acid synthase	4155	100	83
group_2770	siaA	UDP-N-acetylglucosamine 2-epimerase	4014	99	87
group_7987		hypothetical protein	4058	98	84
neuA_2		N-acylneuraminate cytidylyltransferase	4059	96	77
**ERR1163713**	**XIV**				
pglH_1		GalNAc-alpha-(1->4)-GalNAc-alpha-(1->3)-diNAcBac-PP-undecapreno	4002	69	73
gspA		General stress protein A	4003	N/A	N/A
group_7985	epsH	Putative glycosyltransferase EpsH	4056	36	82
group_7986	cstIII	GT-42 glycosyltransferase	4057	100	82
legI_1		N,N'-diacetyllegionaminic acid synthase	4155	100	83
group_2770	siaA	UDP-N-acetylglucosamine 2-epimerase	4014	99	87
group_7987		hypothetical protein	4058	98	84
neuA_2		N-acylneuraminate cytidylyltransferase	4059	96	77
**SRR5152313**	**incomplete**				
group_10079	epsJ_4	putative glycosyltransferase EpsJ	4188	100	100
group_10078		hypothetical protein	4186	100	100
group_10077	epsJ_3	putative glycosyltransferase EpsJ	4187	100	100
?			?	100	100
group_10088	cstIII	GT-42 glycosyltransferase	4159	100	100
legI_2		N,N'-diacetyllegionaminic acid synthase	4181	100	100
siaA		UDP-N-acetylglucosamine 2-epimerase	4182	100	100
neuA		N-acylneuraminate cytidylyltransferase	4183	100	100
group_10086		hypothetical protein	4184	100	100

Group 4					
**Gene**	**Non-unique Gene name**	**Annotation**	**Orthologue**	**coverage**	**%nt id to *C. jejuni***
**ERR1163434**	**XV**				
group_5652	epsJ_1	putative glycosyltransferase EpsJ	4009	98	81
group_7342		hypothetical protein	4010	98	87
group_7343	epsH	Putative glycosyltransferase EpsH	4011	99	84
group_7344	cstII	GT-42 glycosyltransferase	4012	97	87
legI_1		N,N'-diacetyllegionaminic acid synthase	4155	94	84
group_2770	siaA	UDP-N-acetylglucosamine 2-epimerase	4014	100	87

Color code

- *C. coli* clade 1
- *C. coli* clade 2
- *C. coli* clade 3
- GT42 group 2 cstIII
- GT42 group 4 cstII
- GT42 group 5 cstV
- GT42 group 6 cstVI
- GT42 group 7 cstIV
- Sialic acid biosynthesis genes
- Capsule genes

N/A: No significant similarity found

group_7345	neuA	N-acylneuraminate cytidylyltransferase	4015	N/A	N/A
**ERR084011**	**XVI**				
group_5814	pglH_1	GalNAc-alpha-(1->4)-GalNAc-alpha-(1->3)-diNAcBac-PP-undecapreno	4164	100	100
group_5815	epsH	Putative glycosyltransferase EpsH	4163/4172	100	97/99
group_5816	epsJ_2	putative glycosyltransferase EpsJ	4162	100	97
epsH_1		Putative glycosyltransferase EpsH	4161	99	99
group_6953	cstII	GT-42 glycosyltransferase	4149	100	100
group_5817	legI_1	N,N'-diacetyllegionaminic acid synthase	4150	100	100
group_4682	siaA	UDP-N-acetylglucosamine 2-epimerase	4051	100	100
group_4496	neuA	N-acylneuraminate cytidylyltransferase	4052	100	100
group_2414	cat	Streptogramin A acetyltransferase	4168	100	100
**ERR593723**	**XVII**				
group_5351	epsJ_3	putative glycosyltransferase EpsJ	4172	100	99
group_5816	epsJ_2	putative glycosyltransferase EpsJ	4162	100	97
epsH_1		Putative glycosyltransferase EpsH	4161	99	99
group_4171	cstII	GT-42 glycosyltransferase	4153	100	99
group_5817	legI_1	N,N'-diacetyllegionaminic acid synthase	4150	100	100
group_4682	siaA	UDP-N-acetylglucosamine 2-epimerase	4051	100	100
group_4496	neuA	N-acylneuraminate cytidylyltransferase	4052	100	100
vatD		Streptogramin A acetyltransferase	4171	100	99
group_2770	siaA	UDP-N-acetylglucosamine 2-epimerase	4014		
legI_1		N,N'-diacetyllegionaminic acid synthase	4155		
rkpK		UDP-glucose 6-dehydrogenase	4042		
group_4438		UDP-glucose 4-epimerase	4041		
galE_2		UDP-glucose 4-epimerase	4076		
rfbD		UDP-galactopyranose mutase	4075		
neuA_2		N-acylneuraminate cytidylyltransferase	4059		
group_2415	epsJ_1	putative glycosyltransferase EpsJ	4169		
**SRR4242504**	**XVIII**				
group_5351	epsJ_3	putative glycosyltransferase EpsJ	4172	100	99
group_5816	epsJ_2	putative glycosyltransferase EpsJ	4162	100	97
epsH_1		Putative glycosyltransferase EpsH	4161	99	99
group_4169	cstII	GT-42 glycosyltransferase	4158	100	99
group_5817	legI_1	N,N'-diacetyllegionaminic acid synthase	4150	100	100
group_4682	siaA	UDP-N-acetylglucosamine 2-epimerase	4051	100	100
group_4700		hypothetical protein	4179	100	99
group_4496	neuA	N-acylneuraminate cytidylyltransferase	4052	100	100

vatD		Streptogramin A acetyltransferase	4171	100	99
group_2414	cat	Streptogramin A acetyltransferase	4168	100	99
group_2770	siaA	UDP-N-acetylglucosamine 2-epimerase	4014		
legI_1		N,N'-diacetyllegionaminic acid synthase	4155		
rkpK		UDP-glucose 6-dehydrogenase	4042		
group_4438		UDP-glucose 4-epimerase	4041		
galE_2		UDP-glucose 4-epimerase	4076		
rfbD		UDP-galactopyranose mutase	4075		
neuA_2		N-acylneuraminate cytidylyltransferase	4059		
**SRR4453631**	**XIX**				
group_6437	pglH_1	GalNAc-alpha-(1->4)-GalNAc-alpha-(1->3)-diNAcBac-PP-undecapreno	4180	100	85
group_5351	epsJ_3	putative glycosyltransferase EpsJ	4172	100	99
group_5816	epsJ_2	putative glycosyltransferase EpsJ	4162	100	97
epsH_1		Putative glycosyltransferase EpsH	4161	99	99
group_4171	cstII	GT-42 glycosyltransferase	4153	100	99
group_5817	legI_1	N,N'-diacetyllegionaminic acid synthase	4150	100	100
group_4682	siaA	UDP-N-acetylglucosamine 2-epimerase	4051	100	100
group_4496	neuA	N-acylneuraminate cytidylyltransferase	4052	100	100
vatD		Streptogramin A acetyltransferase	4171	100	99
**ERR593755**	**XX**				
group_5351	epsJ_3	putative glycosyltransferase EpsJ	4172	100	99
group_4168	cstII	GT-42 glycosyltransferase	4158	100	99
group_5817	legI_1	N,N'-diacetyllegionaminic acid synthase	4150	100	100
group_4682	siaA	UDP-N-acetylglucosamine 2-epimerase	4051	100	100
group_4496	neuA	N-acylneuraminate cytidylyltransferase	4052	100	100
vatD		Streptogramin A acetyltransferase	4171	100	99
**SRR5794756**	**XXI**				
group_10251	epsJ_1	putative glycosyltransferase EpsJ	4197	67	91
group_10252		hypothetical protein	4196	100	100
group_10253	epsJ_2	putative glycosyltransferase EpsJ	4195	100	100
group_4169	cstII	GT-42 glycosyltransferase	4158	100	99
group_5817	legI_1	N,N'-diacetyllegionaminic acid synthase	4150	100	100
group_4682	siaA	UDP-N-acetylglucosamine 2-epimerase	4051	100	100
group_4700		hypothetical protein	4179	100	99
group_4496	neuA	N-acylneuraminate cytidylyltransferase	4052	100	100
vatD		Streptogramin A acetyltransferase	4171	100	99

**SRR3215130**	**XXII**				
group_5814	pglH_1	GalNAc-alpha-(1->4)-GalNAc-alpha-(1->3)-diNAcBac-PP-undecapreno	4164	100	100
group_5351	epsJ_3	putative glycosyltransferase EpsJ	4172	100	99
group_5816	epsJ_2	putative glycosyltransferase EpsJ	4162	100	97
epsH_1		Putative glycosyltransferase EpsH	4161	99	99
group_4169	cstII	GT-42 glycosyltransferase	4158	100	99
group_5817	legI_1	N,N'-diacetyllegionaminic acid synthase	4150	100	100
group_4682	siaA	UDP-N-acetylglucosamine 2-epimerase	4051	100	100
group_4700		hypothetical protein	4179	100	99
group_4496	neuA	N-acylneuraminate cytidylyltransferase	4052	100	100
group_2414	cat	Streptogramin A acetyltransferase	4168	100	99
**ERR1163833, SRR2920321**	**XXIII**				
group_5920	cstII	GT-42 glycosyltransferase	4132	98	87
legI_1		N,N'-diacetyllegionaminic acid synthase	4155	94	84
group_2770	siaA	UDP-N-acetylglucosamine 2-epimerase	4014	100	87
group_5314	neuA	N-acylneuraminate cytidylyltransferase	4131/4156	91/NA	77/NA
group_8887	vatD	Streptogramin A acetyltransferase	4130/4173	100	85/89

Group 4 and 5

Gene	Non-unique Gene name	Annotation	Orthologue	coverage	%nt id to *C. jejuni*
**SRR1975100**	**XXIV**				
group_9352	pglH_1	GalNAc-alpha-(1->4)-GalNAc-alpha-(1->3)-diNAcBac-PP-undecapreno	4175	99	78
group_5160	cstV	GT-42 glycosyltransferase	4139	N/A	N/A
group_1515	legI_1	N,N'-diacetyllegionaminic acid synthase	4013	N/A	N/A
group_5920	cstII	GT-42 glycosyltransferase	4132	99	88
legI_1		N,N'-diacetyllegionaminic acid synthase	4155	100	83
group_2770	siaA	UDP-N-acetylglucosamine 2-epimerase	4014	100	86
group_5314	neuA	N-acylneuraminate cytidylyltransferase	4131	91	77
group_8887	vatD	Streptogramin A acetyltransferase	4130	100	85

Group 5

Gene	Non-unique Gene name	Annotation	Orthologue	coverage	%nt id to *C. jejuni*
**ERR1163655**	**IX**				
group_5159	pglH_2	GalNAc-alpha-(1->4)-GalNAc-alpha-(1->3)-diNAcBac-PP-undecapreno	4138	95	78
group_5160	cstV	GT-42 glycosyltransferase	4139	N/A	N/A
group_1515	legI_1	N,N'-diacetyllegionaminic acid synthase	4013	99	84
group_2770	siaA	UDP-N-acetylglucosamine 2-epimerase	4014	100	87
group_4585	neuA	N-acylneuraminate cytidylyltransferase	4146	N/A	N/A
group_8111	gspA	General stress protein A	4147	N/A	N/A

**ERR1163885, ERR1163441, ERR1163615, ERR1163639, ERR1163676, ERR1163717, ERR1163831**			**XXV**		
group_5159	pglH_2	GalNAc-alpha-(1->4)-GalNAc-alpha-(1->3)-diNAcBac-PP-undecapreno	4138	95	78
group_5160	cstV	GT-42 glycosyltransferase	4139	N/A	N/A
group_1515	legI_1	N,N'-diacetyllegionaminic acid synthase	4013	99	84
group_2770	siaA	UDP-N-acetylglucosamine 2-epimerase	4014	100	87
group_4585	neuA	N-acylneuraminate cytidylyltransferase	4146/4148	N/A 95	N/A 78
**ERR1163416, ERR1163494, ERR1163599, ERR1163863**		**XXVI**			
group_5159	pglH_2	GalNAc-alpha-(1->4)-GalNAc-alpha-(1->3)-diNAcBac-PP-undecapreno	4138	95	78
group_5160	cstV	GT-42 glycosyltransferase	4139	N/A	N/A
group_1515	legI_1	N,N'-diacetyllegionaminic acid synthase	4013	99	84
group_7292	siaA	UDP-N-acetylglucosamine 2-epimerase	4140	82	88
group_7293		hypothetical protein	4141	N/A	N/A
group_7294		hypothetical protein	4142	97	78
group_7295	neuA	N-acylneuraminate cytidylyltransferase	4143	N/A	N/A

**Group 6**					
**Gene**	**Non-unique Gene name**	**Annotation**	**Orthologue**	**coverage**	**%nt id to *C. jejuni***
**SRR5005386**	**III**				
pglH_1		GalNAc-alpha-(1->4)-GalNAc-alpha-(1->3)-diNAcBac-PP-undecapreno	4002	69	73
gspA		General stress protein A	4003	N/A	N/A
group_5761	epsJ_1	putative glycosyltransferase EpsJ	4004	35	84
group_2111		hypothetical protein	4211	N/A	N/A
group_3997	cstVI	GT-42 glycosyltransferase	4127/cst-F	N/A	N/A
**ERR1163830**	**XXVII**				
group_8851	epsJ_3	putative glycosyltransferase EpsJ	4129	66	75
group_6191		hypothetical protein	4067	N/A	N/A
group_4660	epsJ_2	putative glycosyltransferase EpsJ	4068	97	80
group_8850		hypothetical protein	4069	N/A	N/A
group_8256		hypothetical protein	4070	N/A	N/A
group_4000	cstVI	GT-42 glycosyltransferase	4200	N/A	N/A
**ERR1163806, ERR1163854**	**XXVIII**				
group_6190	epsJ_1	putative glycosyltransferase EpsJ	4066	91	76
group_6191		hypothetical protein	4067	N/A	N/A
group_4660	epsJ_2	putative glycosyltransferase EpsJ	4068	97	80
tagE		putative poly(glycerol-phosphate) alpha-glucosyltransferase	4124	N/A	N/A
group_8755	epsJ_3	putative glycosyltransferase EpsJ	4125	40	83

group_2110		hypothetical protein	4126	N/A	N/A
group_3997	cstVI	GT-42 glycosyltransferase	4127	N/A	N/A
group_2770	siaA	UDP-N-acetylglucosamine 2-epimerase	4014		
legI_1		N,N'-diacetyllegionaminic acid synthase	4155		
group_4658	cstI	hypothetical protein	4157		

**Group 7**					
**Gene**	**Non-unique Gene name**	**Annotation**	**Orthologue**	**coverage**	**%nt id to *C. jejuni***
**ERR1163758**	**II**				
group_7713	epsJ_1	putative glycosyltransferase EpsJ	4201	N/A	N/A
group_7712		hypothetical protein	4208	N/A	N/A
group_2667		hypothetical protein	4209	N/A	N/A
group_6160	cstIV	GT-42 glycosyltransferase	4210	N/A	N/A
group_7711		hypothetical protein	4202	N/A	N/A
cptA		Phosphoethanolamine transferase CptA	4203	N/A	N/A
group_7710		hypothetical protein	4204	N/A	N/A
group_4620		hypothetical protein	4205	N/A	N/A
group_6063		hypothetical protein	4206	N/A	N/A
group_3722		hypothetical protein	4207	N/A	N/A
group_4658		hypothetical protein	4157		
group_7183	neuA	N-acylneuraminate cytidylyltransferase	4156		
legI_1		N,N'-diacetyllegionaminic acid synthase	4155		
group_2770	siaA	UDP-N-acetylglucosamine 2-epimerase	4014		
**ERR1163726**	**XXIX**				
group_5303	epsJ_1	putative glycosyltransferase EpsJ	4060	67	76
group_4659		hypothetical protein	4061	N/A	N/A
group_8389		hypothetical protein	4083	N/A	N/A
group_6184	cstIV	GT-42 glycosyltransferase	4063	N/A	N/A
eptC		Phosphoethanolamine transferase EptC	4084	N/A	N/A
group_2770	siaA	UDP-N-acetylglucosamine 2-epimerase	4014		
legI_1		N,N'-diacetyllegionaminic acid synthase	4155		
group_4585	neuA	N-acylneuraminate cytidylyltransferase	4146		
**ERR1163788, SRR1975124, ERR1163731, SRR3214492**				**30**	
group_5303	epsJ_1	putative glycosyltransferase EpsJ	4060	67	76
group_4659		hypothetical protein	4061	N/A	N/A
group_2671		hypothetical protein	4062	N/A	N/A

group_6184	cstIV	GT-42 glycosyltransferase	4063	N/A	N/A
eptC		Phosphoethanolamine transferase EptC	4084	N/A	N/A
**ERR1163754**	**XXXI**				
group_8560	epsJ_1	putative glycosyltransferase EpsJ	4101	91	77
group_4659		hypothetical protein	4061	N/A	N/A
group_2671		hypothetical protein	4062	N/A	N/A
group_6184	cstIV	GT-42 glycosyltransferase	4063	N/A	N/A
eptC		Phosphoethanolamine transferase EptC	4084	N/A	N/A
group_2770	siaA	UDP-N-acetylglucosamine 2-epimerase	4014		
legI_1		N,N'-diacetyllegionaminic acid synthase	4155		
group_4658		hypothetical protein	4157		
group_5314	neuA	N-acylneuraminate cytidylyltransferase	4131		
**ERR1163708 and SRR3214482**	**XXXII**				
group_5303	epsJ_1	putative glycosyltransferase EpsJ	4060	67	76
group_4659		hypothetical protein	4061	N/A	N/A
group_2671		hypothetical protein	4062	N/A	N/A
group_6184	cstIV	GT-42 glycosyltransferase	4063	N/A	N/A
pssM		Exopolysaccharide glucosyl ketal-pyruvate-transferase	4064	N/A	N/A
group_8235		hypothetical protein	4065	43	81
**ERR1163818, ERR1163835**		**XXXIII**			
group_5303	epsJ_1	putative glycosyltransferase EpsJ	4060	67	76
group_4659		hypothetical protein	4061	N/A	N/A
group_2671		hypothetical protein	4062	N/A	N/A
group_6184	cstIV	GT-42 glycosyltransferase	4063	N/A	N/A
eptC		Phosphoethanolamine transferase EptC	4084	N/A	N/A
pssM		Exopolysaccharide glucosyl ketal-pyruvate-transferase	4064	N/A	N/A
group_8235		hypothetical protein	4065	43	81
**SRR1975129**	**XXXIV**				
group_5303	epsJ_1	putative glycosyltransferase EpsJ	4060	67	76
group_4659		hypothetical protein	4061	N/A	N/A
group_2671		hypothetical protein	4062	N/A	N/A
group_6184	cstIV	GT-42 glycosyltransferase	4063	N/A	N/A
eptC		Phosphoethanolamine transferase EptC	4084	N/A	N/A
eptC_2		Phosphoethanolamine transferase EptC	4176	N/A	N/A
group_9399		hypothetical protein	4177	N/A	N/A

group_2770	siaA	UDP-N-acetylglucosamine 2-epimerase	4014		
legI_1		N,N'-diacetyllegionaminic acid synthase	4155		
**ERR1163785**	**XXXV**				
group_8590	epsJ_2	putative glycosyltransferase EpsJ	4105	66	76
group_6243		hypothetical protein	4106	N/A	N/A
group_6244		hypothetical protein	4107	N/A	N/A
group_8591	cstIV	GT-42 glycosyltransferase	4108	N/A	N/A
eptC_1		Phosphoethanolamine transferase EptC	4109	N/A	N/A
group_8593	eptC_2	Phosphoethanolamine transferase EptC	4110	N/A	N/A
group_4522	fdtB	dTDP-3-amino-3,6-dideoxy-alpha-D-galactopyranose transaminase	4018	N/A	N/A
group_8594	wfgD	UDP-Glc:alpha-D-GlcNAc-diphosphoundecaprenol beta-1,3-glucosyltr	4111	N/A	N/A
group_8595		hypothetical protein	4112	N/A	N/A
group_8596	epsE	Putative glycosyltransferase EpsE	4113	N/A	N/A
group_6229		hypothetical protein	4097	N/A	N/A
group_8529	lacA_1	Galactoside O-acetyltransferase	4098	N/A	N/A
fdtA		TDP-4-oxo-6-deoxy-alpha-D-glucose-3,4-oxoisomerase	4078	N/A	N/A
oatWY		Polysialic acid O-acetyltransferase	4077	N/A	N/A
rfbB		dTDP-glucose 4,6-dehydratase	4026	N/A	N/A
rmlA		Glucose-1-phosphate thymidylyltransferase	4027	N/A	N/A

plementary Table 6

Genome (Acc. Number)	MLST Sequence type	ReMatCh results for GT-42
ERR221394	19	*cstIV*
ERR221300	48	*cstIV*
SRR5170912	459	*cstIV*
SRR2976823	459	*cstIV*
SRR3583303	459	*cstIV*
SRR4018132	459	*cstIV*
SRR2727977	459	*cstIV*
SRR3066579	459	*cstIV*
SRR5808785	459	*cstIV*
SRR5821279	459	*cstIV*
SRR3953110	459	*cstIV*
SRR3611765	459	*cstIV*
SRR5297422	459	*cstIV*
SRR5209908	459	*cstIV*
SRR3543495	459	*cstIV*
ERR1906140	1286	*cstIV*
ERR1906133	1286	*cstIV*
ERR1906188	1316	*cstIV*
ERR226168	2568	*cstIV*
ERR152270	2568	*cstIV*
SRR3402101	4486	*cstIV*
ERR349076	6564	*cstIV*
ERR349092	7278	*cstIV*
ERR1025561	7546	*cstIV*
SRR5230154	-	*cstIV*
SRR5297186	-	*cstIV*
SRR4175504	-	*cstIV*
SRR4022328	-	*cstIV*
SRR5222006	-	*cstIV*
ERR349072	-	*cstIV*
SRR4022329	-	*cstIV*
SRR3196134	-	*cstIV*
SRR3490037	-	*cstIV*
ERR222680	-	*cstIV*
SRR3619952	-	*cstIV*
ERR349126	-	*cstIV*
SRR3490036	-	*cstIV*
SRR3882939	-	*cstIV*
SRR5925242	-	*cstIV*
ERR355978	-	*cstIV*
ERR196590	19	*cstVI*
ERR211364	21	*cstVI*
ERR152288	45	*cstVI*
ERR196398	61	*cstVI*
ERR196702	1044	*cstVI*
ERR152190	2568	*cstVI*
ERR128771	-	*cstVI*
ERR211196	-	*cstVI*
ERR211199	-	*cstVI*
ERR193035	-	*cstVI*
ERR084058	3029	*cstVII*
ERR1815190	-	*cstVII*

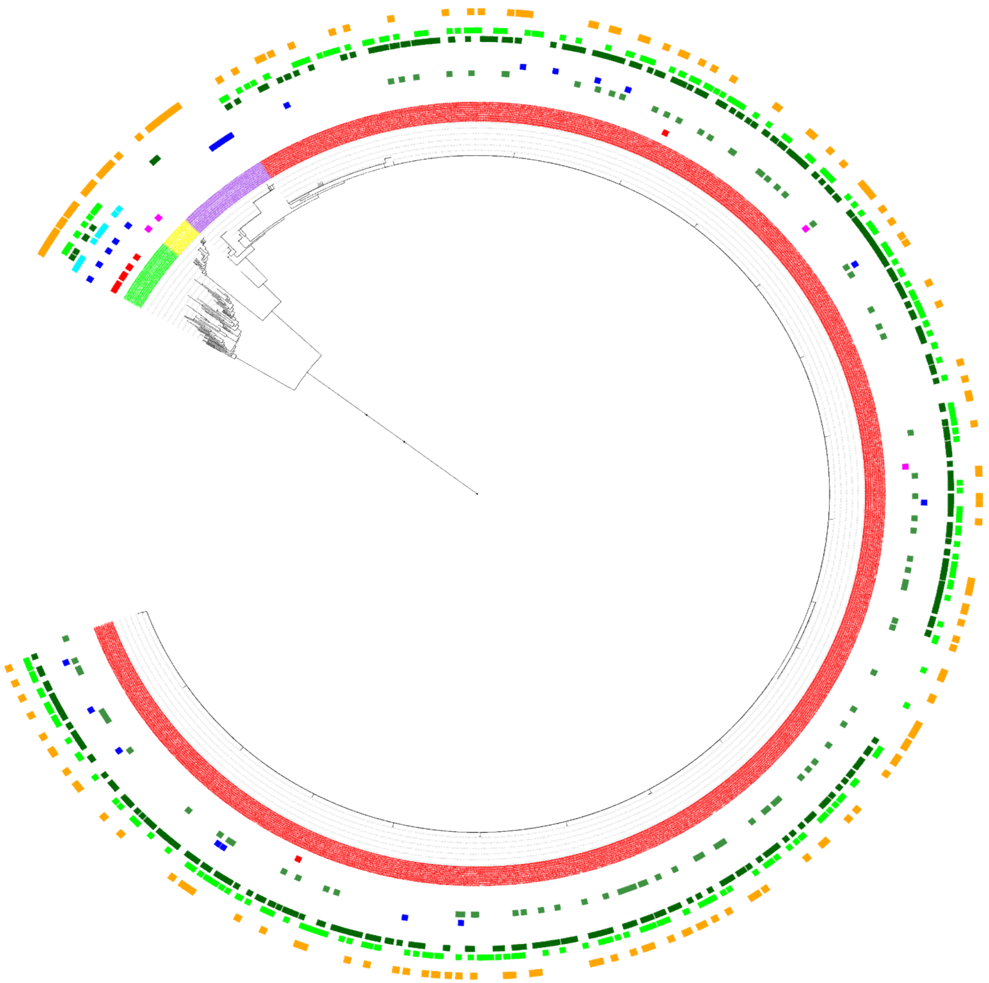

Figure S1. Evolutionary analyses of 2553 C. coli strains based on atpA conducted in MEGA7 and the evolutionary history was inferred using the Minimum Evolution method calculating the distance using Maximum Composite Likelihood. Colour of the leaf correspond of the C. coli clades as defined by Sheppard and colleagues1, and inferred using hierBAPS2: Clade 1a, red; Clade 1b and 1c, violet; Clade 2, yellow; Clade 3, green. Circles from in to out, positive ReMatCh results for: GT42 Group 1 (cstI), red; GT42 Group 2 (cstIII), pink; GT42 Group 3 (cstVII), turquoise; GT42 Group 4 (cstII), blue; GT42 Group 5 (cstV), cyan; GT42 Group 6 (cstVI), green; GT42 Group 7 (cstIV), light green; neuB, orange.

REFERENCES

1 Sheppard, S. K. *et al.* Progressive genome-wide introgression in agricultural Campylobacter coli. *Mol. Ecol.* **22,** 1051–1064 (2013).

2 Cheng, L., Connor, T. R., Sirén, J., Aanensen, D. M. & Corander, J. Hierarchical and Spatially Explicit Clustering of DNA Sequences with BAPS Software. *Mol. Biol. Evol.* **30,** 1224–1228 (2013).
